# Mental Scene and Memory Construction and the Hippocampus

**DOI:** 10.1002/hipo.70136

**Published:** 2026-09-28

**Authors:** Edmund T. Rolls

**Affiliations:** ^1^ Oxford Centre for Computational Neuroscience Oxford UK; ^2^ Department of Computer Science University of Warwick Coventry UK

**Keywords:** autobiographical memory, episodic memory, hippocampus, parahippocampal scene area, prospective memory, scene construction, scene representations, spatial view cells

## Abstract

A neurocomputational theory of scene and memory construction during memory recall, prospective thinking, and autobiographical memory is described. The theory describes the nature of the representation of scenes using spatial view cells in a continuous attractor network in the medial parahippocampal cortex, and how the location in the scene being represented can be shifted by self‐motion eye position update even when the scene is not visible. The theory builds on the evidence for humans for a ventromedial visual cortical scene pathway via the prostriate cortex and ventromedial visual cortical regions to the medial parahippocampal scene (or place) area where scenes are represented. The approach builds on a theory about how visual features in scenes are used along this pathway to build a continuous attractor network representation of scenes in the medial parahippocampal cortex that represents the relative spatial locations of features or landmarks in the scene. The theory shows how episodic memory can be built in an attractor network in hippocampal CA3 that learns associations between the location in a scene (“where”), the object or person at that location in the scene (“what”), and the reward/affective value conveyed to the hippocampus from the orbitofrontal cortex in part via the ventromedial prefrontal cortex and posterior cingulate cortex. The theory of scene construction then shows how backprojections from hippocampal CA1 via the entorhinal cortex to the medial parahippocampal cortex can recall or construct the scene representation in the medial parahippocampal cortex, the parahippocampal scene (or place) area; and how whole memories including visual imagery of objects, rewards, and their locations in scenes can be constructed by iterative neocortical‐hippocampal‐neocortical processing.

## 1 Introduction

Mental scene construction refers to the neurocognitive processes involved in mentally generating and maintaining complex and coherent scenes or events (Hassabis et al. 2007; Maguire and Mullally 2013; Mullally and Maguire 2013). The term construction refers not to scene perception per se, but instead to the recall of scenes, or autobiographical memories, or imagined future thinking, all of which typically involve scenes. Mental scene construction is important not only for understanding how human memory works, but more generally for understanding how mental models work (Duncan 2025), and impairments in such mentalizing in some mental disorders and in aging (Acevedo‐Molina et al. 2023; Conti et al. 2024).

One aim of this paper is to describe the construction of scenes during episodic memory recall, but also from autobiographical memory, and imagining scenes in for example prospective memory. A second aim is to extend this to how whole memories with semantic content can be constructed starting with a recall cue. The plan of the paper is to describe in Sections 2, 9 the empirical research and previous theoretical work that provide the foundation for the new theories, and to present the new theories in Section 10. First, in Section 2 the brain regions involved in scene construction compared to those in scene perception are described; then the scene pathways that lead to the hippocampus which is a key brain region involved in scene construction (Section 3); then the face, object, and reward pathways to the hippocampus that are involved in the recall of memories (Section 4); and then how scenes are represented in the brain (Section 5); and how scene representations in the brain are built (Section 6). To continue towards understanding how scenes are constructed from memory, it is then necessary to consider the way in which the hippocampus stores information in memory (Section 7), and how the hippocampus recalls information from memory back to the neocortex (Section 8). Because long‐term semantic knowledge (e.g., about people and objects) can be included in these memories that are stored and reconstructed, the connectivity between anterior temporal lobe sematic systems and the hippocampal memory system is described (Section 9). All of this evidence is necessary to describe a theory of scene and memory construction (Section 10), including how the hippocampus and related systems are involved in scene construction during recall and future imagining; the extent to which the ventromedial prefrontal cortex (vmPFC) is involved in the scene construction processes, as has been suggested (Bonnici et al. 2012; Bonnici and Maguire 2018; McCormick et al. 2018, 2020; De Luca et al. 2019); and how full memories with semantic content are constructed.

The evidence comes from neurophysiology, functional imaging, and neuropsychology, but importantly extends to a computational neuroscience approach, for by understanding *what* computations are performed by brain regions and *how* they are performed, we can develop a well‐founded understanding of how cognitive and other brain functions are performed (Rolls 2021c, 2023c; Wang 2025; Rolls 2026b).

We start by considering the brain systems that are involved in scene construction, and how they compare to those involved in scene perception.

## 2 Scene Perception vs. Scene Construction From Memory

Key brain regions involved in scene construction are the retrosplenial place or scene area (also known as the medial place area) (Silson, Gilmore, et al. 2019; Silson, Steel, et al. 2019; Steel et al. 2021); the parahippocampal place or scene area (Steel et al. 2021); and the hippocampus (Zeidman et al. 2015; Zeidman and Maguire 2016) (the retrosplenial place or scene area is not in region RSC, but is in the ProStriate Cortex (ProS) and POS1 regions (Silson et al. 2016; Silson, Gilmore, et al. 2019; Rolls, Feng, and Zhang 2024), as shown in Figure 2) (the term scene area is preferred to place area, because it is viewing scenes “out there” that activates these regions in primates including humans, not the specific place where the individual is located, which is an important distinction compared to rodents, in which it is the place where the rodent is located that is relevant (Rolls 2026a, 2027)). In all three cases, it is the anterior parts of the regions activated during scene viewing (as shown by fMRI (Rolls, Feng, and Zhang 2024)) that are activated during scene construction (in for example the recall of scenes, of familiar scenes, and of autobiographical memories) (Zeidman et al. 2015; Zeidman and Maguire 2016; Silson, Steel, et al. 2019; Steel et al. 2021; Kidder et al. 2025). These three brain regions involved in viewing scenes are labeled in Figure 1 on a view of the Human Connectome Project Multimodal Parcellation (HCP‐MMP) atlas (Glasser, Coalson, et al. 2016) and its extended version (Huang et al. 2022), which was used for some of the investigations described here, because it uses multimodal information, including cortical myelin, cortical thickness, functional connectivity, and task‐related fMRI to delineate 180 cortical regions in each hemisphere that are potentially different computational modules in the cerebral cortex (Glasser, Coalson, et al. 2016; Rolls 2023c, 2026b). In this atlas, the retrosplenial scene or place area is in the prostriate cortex (ProS) (typically extending to the adjacent POS1) (Steel et al. 2021; Rolls, Feng, and Zhang 2024; Rolls, Zhang, et al. 2024); and the parahippocampal scene or place area is in the medial parahippocampal regions PHA1‐3 and the ventromedial visual regions VMV1‐3 that connect the retrosplenial scene area with the parahippocampal scene area (Steel et al. 2021; Rolls 2024; Rolls, Feng, and Zhang 2024; Rolls, Zhang, et al. 2024) (see Figures 1 and 2). The activations to scenes in the ventromedial cortical “where” scene pathway (including prostriate cortex ProS, the ventromedial visual regions VMV1‐3, and the medial parahippocampal cortex PHA1‐3) from 956 HCP participants using the HCP‐MMP atlas are illustrated in Figure 2, in which they are compared with activations by faces in regions in the ventrolateral visual cortical “what” pathway (including the fusiform face cortex FFC and inferior temporal visual cortex region TE2p leading to the lateral parahippocampal cortex TF) (Rolls, Feng, and Zhang 2024). In addition to these three scene areas, there is an occipital place area (Dilks et al. 2013), and a connected superior parietal region (Kennedy et al. 2024; Yoon et al. 2025) adjacent to V6 which encodes optic flow, that are implicated more in navigational processes such as egomotion than in scene perception (Yoon et al. 2025).

**FIGURE 1:**
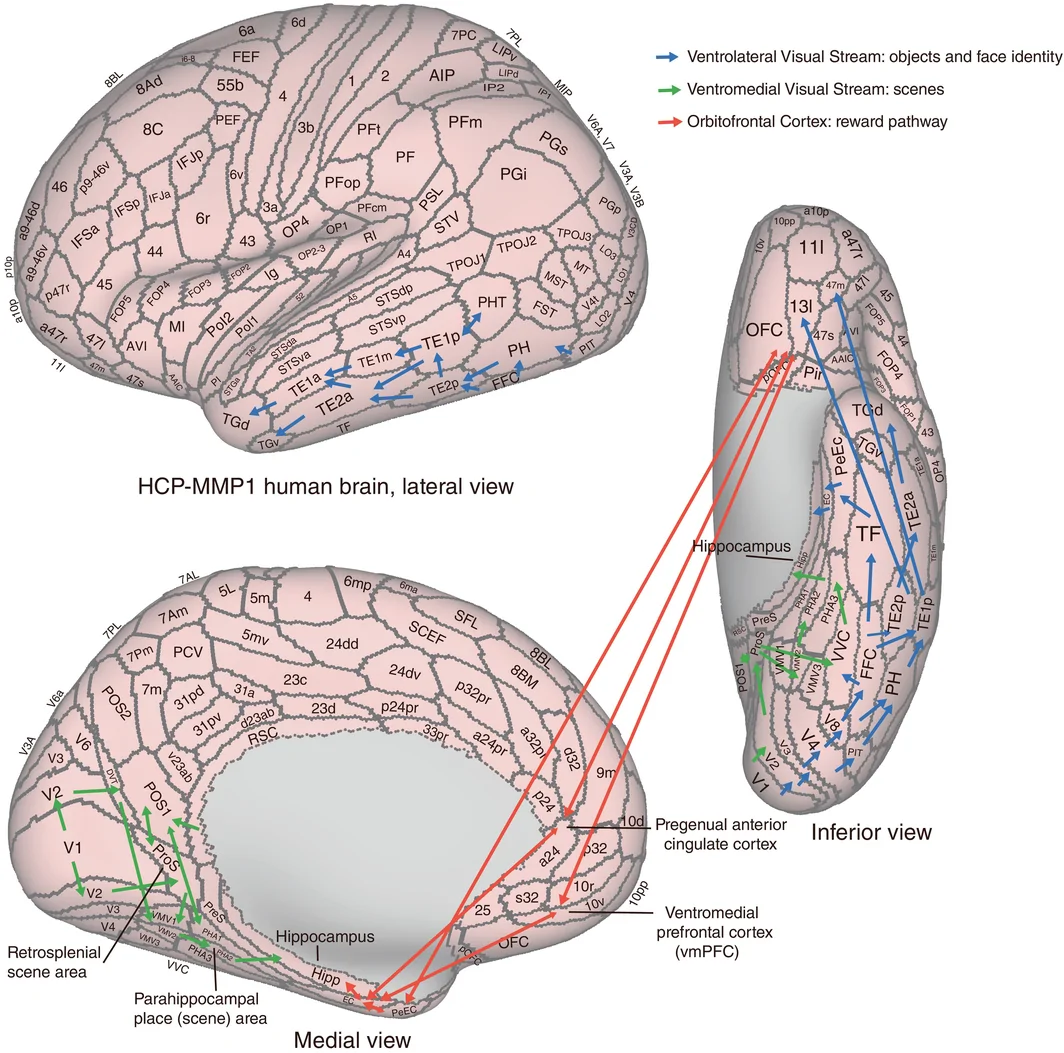
Three key pathways to the human hippocampus for “where,” “what,” and reward. A ventromedial visual cortical pathway for scenes (green arrows) connects from V1 and V2 to the ProStriate Cortex (ProS) where the retrosplenial scene area is located, and then via ventromedial visual cortical regions VMV1‐3 and VVC, and then to the medial parahippocampal gyrus where the parahippocampal place (or, better, scene) area is located, and then in part via the entorhinal cortex to the hippocampus (Huang et al. 2021; Rolls et al. 2022c; Rolls et al. 2023a; Rolls, Yan, et al. 2024). The ventrolateral visual cortical pathway for objects and faces (blue arrows) connects from V1 then V2 and V4 to the fusiform face cortex (FFC) and then via the lateral parahippocampal gyrus (TF), perirhinal cortex (PeEC), and entorhinal cortex (EC) to the hippocampus (Rolls et al. 2023a; Rolls, Deco, et al. 2023). The reward‐related medial orbitofrontal cortex (red arrows) connects especially from OFC and pOFC to the perirhinal cortex, then entorhinal cortex to the hippocampus, with in addition routes via the pregenual anterior cingulate cortex and ventromedial prefrontal cortex (Rolls et al. 2022a). In addition, the lateral orbitofrontal cortex 47 m has similar connectivity to reach the hippocampus (Rolls et al. 2022a) (see Figure 3). The pathways were analyzed with effective connectivity and functional connectivity with fMRI and magnetoencephalography, and diffusion tractography in 171 Human Connectome Project participants. The pathways are shown on the parcellation of the human cortex in the HCP‐MMP atlas (Glasser, Coalson, et al. 2016), and in its extended version HCPex (Huang et al. 2022). The regions are shown on images of the human brain in inflated form with the sulci expanded sufficiently to allow the regions within the sulci to be shown. The abbreviations for each cortical region are provided in Table S1, and the cortical regions are illustrated in Figure S1 (updated from Rolls 2026a).

**FIGURE 2:**
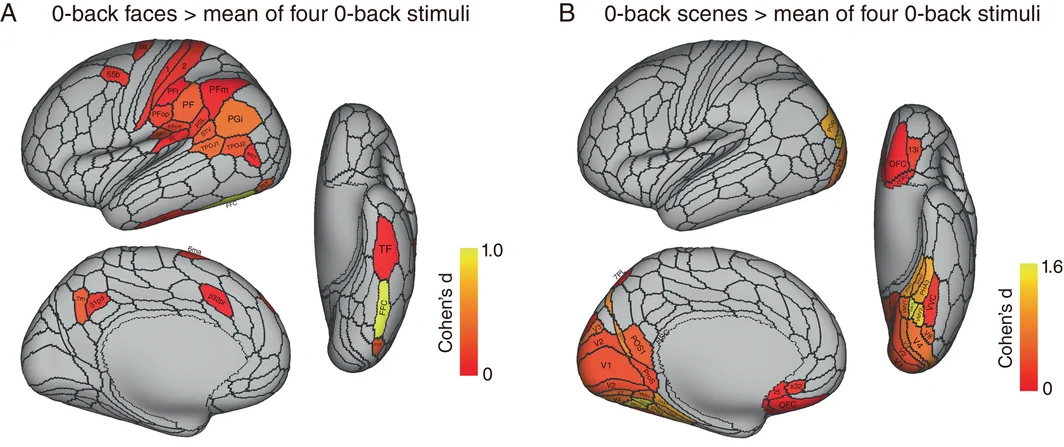
Activations in visual cortical pathways to the human hippocampus. Scenes activate ventromedial cortical regions including the ProStriate Cortex (ProS), POS1, ventromedial cortical regions VMV1‐3, and medial parahippocampal regions PHA1‐3. Faces activate the ventrolateral temporal regions including FFC (fusiform face cortex), lateral parahippocampal TF, and PIT (posterior inferior temporal cortex). The contrasts shown are for the average BOLD signal for faces (A), and scenes (B) compared to the mean of faces, scenes, body parts, and tools in a 0‐back working memory task, after Bonferroni correction (*α* = 0.05) across 956 Human Connectome Project participants. The effect size as indicated by Cohen's *d* is indicated. The activations are shown in red to yellow. The abbreviations for each cortical region in the HCP Multimodal Parcellation (Glasser, Coalson, et al. 2016) are provided in Table S1, and the cortical regions are illustrated in Figure S1. The retrosplenial scene area is in ProS and POS1, and cortical region RSC was not selectively activated by scenes. The parahippocampal scene area (or PPA) is in PHA1‐3 extending to VMV1‐3 (adapted from Rolls, Feng, and Zhang 2024). The corresponding activations in the right hemisphere are shown in that paper; and laterality and sex differences are also available (Rolls et al. 2026; Zhang et al. 2026b).

## 3 Brain Regions and Pathways Involved in Scene Perception That Provide Scene Inputs to the Hippocampus

It has long been known that viewing scenes can activate a parahippocampal “place” area and a retrosplenial “place” area (Epstein and Kanwisher 1998; Epstein 2005; Epstein and Julian 2013; Epstein and Baker 2019), with the regions now well defined in the HCP‐MMP atlas (Steel et al. 2021; Rolls, Feng, and Zhang 2024; Rolls, Zhang, et al. 2024; Rolls et al. 2026), and noted above (see Figures 1 and 2). More recently, it has been possible to trace in the human brain a “ventromedial cortical where scene pathway” as illustrated in green in Figure 1 (Rolls 2024, 2026a), using effective and functional connectivity with fMRI (Rolls et al. 2023a) and magnetoencephalography (Rolls, Yan, et al. 2024), and using diffusion tractography (Rolls et al. 2023a), as described next.

The visual cortical ventromedial “Where” scene pathway has connectivity from V1 to the prostriate cortex (ProS) which with POS1 is where the retrosplenial scene or place area is located, via cortical ventromedial visual regions VMV1‐3 and VVC to the medial parahippocampal gyrus PHA1‐3 which is where the parahippocampal scene or place area is located, and then via the medial entorhinal cortex to the hippocampus (Huang et al. 2021; Rolls et al. 2022c; Rolls et al. 2023a; Rolls 2024; Rolls, Yan, et al. 2024; Rolls and Turova 2025) (Figure 1). This ventromedial cortical pathway is activated selectively by scenes, relative to faces, tools, and body parts (Rolls, Feng, and Zhang 2024) (Figure 2). This pathway is also activated selectively during an episodic memory task in which the location of an object or a reward in a scene is stored, and then retrieved from memory (Rolls, Zhang, et al. 2024). A feature of this pathway as described later in Section 6 is that it is a ventral visual cortical stream pathway for “Where,” that functions computationally by building feature combinations for spatial features that are close together in scenes, and then linking these features using associative learning in the recurrent collateral connections between the neurons to build a continuous attractor network representation of a scene (Rolls 2025a). Anchoring the spatial scene representations provided by spatial view cells to the features in part of a spatial scene makes this an allocentric, world‐based, representation (Feigenbaum and Rolls 1991; Rolls et al. 1997, 1998; Georges‐François et al. 1999; Rolls 2025a). Gain modulation by gaze direction is used to link visual fixation patches across saccades (Rolls 2025a).

## 4 Pathways for “What” and Reward Inputs to the Hippocampus

The classical ventral visual cortical stream, now termed a visual ventrolateral “what” cortical pathway, for objects and faces is shown in blue in Figure 1 (Rolls 2024). The pathway connects from V1 via V2, V3, and V4 to the fusiform face cortex FFC, and from there to the inferior temporal visual cortex regions TE1p and TE2p, and from there to the lateral parahippocampal cortex region TF, which then connects via perirhinal cortex (PeEc) and entorhinal cortex (EC) to the hippocampus (Rolls et al. 2023a; Rolls, Deco, et al. 2023). In this path transform‐invariant neurons were discovered that represent faces (Perrett et al. 1982; Rolls 1984, 2025b, 2026b, 2026c) and objects (Booth and Rolls 1998) in macaques. These are important by providing a “What” input to the hippocampus, enabling any episodic memory formed to generalize to transforms including views of the people or objects (Rolls 2021d). This pathway is selectively activated in humans by faces, relative to scenes, tools, and body parts (Rolls, Feng, and Zhang 2024) (Figure 2), and is activated in an episodic memory task for where objects or rewards have been shown in scenes (Rolls, Zhang, et al. 2024).

Another key pathway to the hippocampus again recently traced in humans is from the primate including human orbitofrontal cortex which represents the reward and emotional value of faces, objects, social stimuli and money (Rolls 2019, 2023d, 2026c). These orbitofrontal cortex regions connect partly via the ventromedial prefrontal cortex (vmPFC) and anterior cingulate cortex, then perirhinal cortex and then entorhinal cortex, to the hippocampus (red in Figure 1; and Figure 3), thus providing the reward value inputs to the hippocampus that provide an important component of most episodic memories (Rolls 2022). The orbitofrontal cortex inputs to the hippocampal memory system are implicated in memory consolidation, partly because reward value information may increase the probability that memories are processed more (Rolls 2022). This input from the orbitofrontal cortex for reward value‐related information to reach the hippocampus is remarkably highly developed in humans, and includes routes via the ventromedial prefrontal cortex and the posterior cingulate cortex to the hippocampal memory system (Figure 3) (Rolls et al. 2022a; Rolls 2027). The ventromedial prefrontal cortex is known to be activated by many reward/emotion‐related/stimuli, as is the medial orbitofrontal cortex from which it receives (Grabenhorst and Rolls 2011; Rolls 2022, 2023d; Rolls et al. 2022a).

**FIGURE 3:**
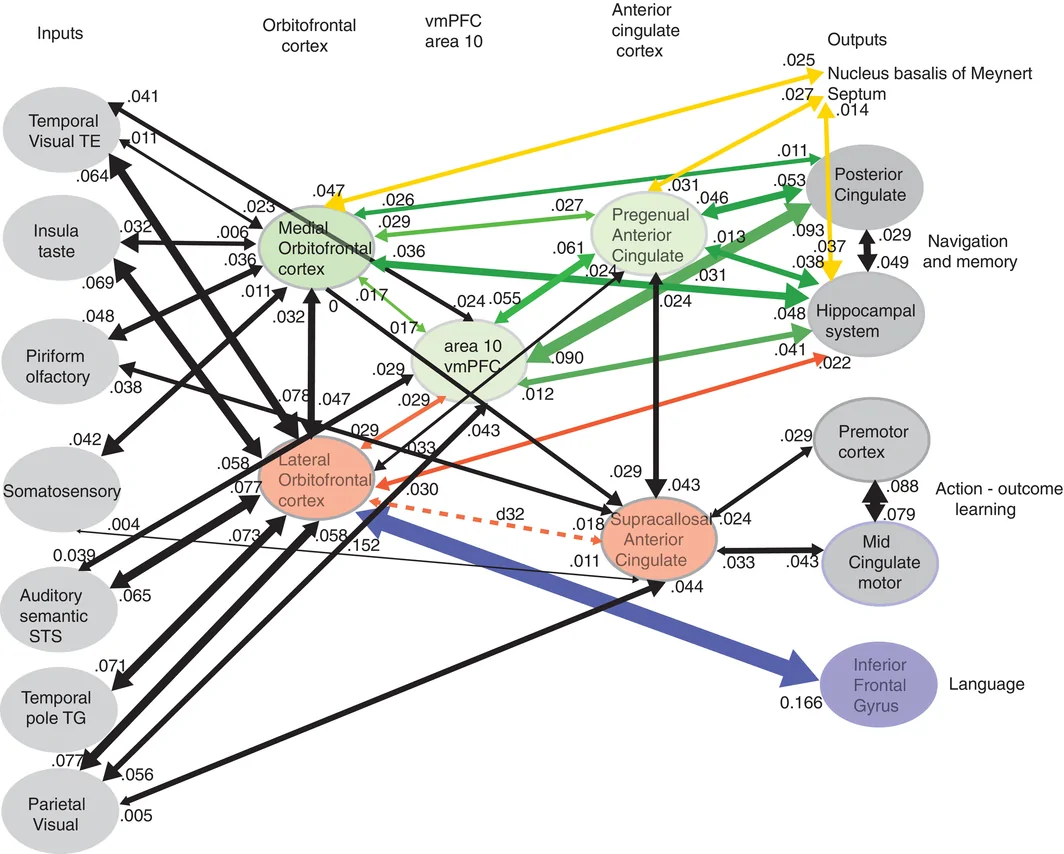
Effective connectivity of the orbitofrontal cortex, vmPFC, and anterior cingulate cortex shown in the middle, with inputs on the left and outputs including to the hippocampal formation via perirhinal and entorhinal cortex on the right. The cortical regions included for each of the 5 central ellipses are defined by Rolls et al. (2022a). The green ellipses are areas that are activated mainly by rewards, and the red ellipses are areas activated mainly by punishers or non‐reward (not receiving an expected reward). The lateral orbitofrontal cortex has connectivity with the supracallosal anterior cingulate cortex via region d32 as shown with the dashed line. The width of the arrows is proportional to the effective connectivity in the highest direction, and the size of the arrows reflects the strength of the effective connectivity in each direction. The effective connectivities shown are for the strongest link where more than one link between regions applies for a group of brain regions. The effective connectivities in yellow are to the basal forebrain nuclei of Meynert which contains cholinergic neurons that project to the neocortex and to the septal nuclei which contain cholinergic neurons that project to the hippocampus. The Somatosensory regions include 5 and parietal PF and PFop, which also connect to the pregenual anterior cingulate but are not shown for clarity; and the Parietal regions include visual parietal regions 7, PGi and PFm. The connectivity with dorsolateral prefrontal cortex is not included here for clarity. Connectivity is shown for the five groups in the center of the figure, and does not include for example connectivity between somatosensory and premotor cortical regions (updated from Rolls et al. 2022a).

These discoveries (Rolls 2025b, 2026c) show that the primate (including human) hippocampus receives information about spatial view locations “out there” in space via a ventromedial “where” visual cortical pathway; and about the face or object that is present at the visually fixated location (Rolls et al. 2003; Aggelopoulos and Rolls 2005) via the ventrolateral “what” visual cortical pathway; and about reward value including face expression and identity (Rolls et al. 2006), and social (Kringelbach and Rolls 2003), and monetary (O'Doherty et al. 2001; Rolls et al. 2020) rewards from the orbitofrontal cortex, partly via the ventromedial prefrontal cortex (Rolls 2019, 2023c, 2025c, 2026b). These pathways to and from the hippocampus are highly developed in primates including especially humans, with no close equivalents in rodents (Rolls 2026a, 2026b, 2027).

## 5 The Representation of Scenes in the Primate Including Human Hippocampus: Spatial View Cells

To understand how scenes can be reconstructed during for example scene or autobiographical memory recall, we need to consider next how scenes are in fact represented in the primate including human brain. The discovery of “spatial view cells” in primates provides a foundation for understanding how scenes are represented in primates including humans (Rolls 2023a, 2026a, 2026b, 2026c, 2027). Indeed, a revolution in the understanding of hippocampal function is now taking place, for evidence is accumulating that many macaque and human hippocampal neurons respond to the spatial location that is being viewed, and are termed spatial view cells, which are very different (Rolls 2026a, 2027) to the hippocampal place cells described in rodents (O'Keefe 1979; Moser et al. 2017), as described next.

The initial discoveries were that some monkey hippocampal neurons respond to some but not other locations “out there” in space on a screen when the monkey is performing an object‐location memory task in which the location of an object on the screen needed to be remembered (Cahusac et al. 1989; Rolls et al. 1989).

We followed up in recordings from hippocampal neurons while macaques were performing a task in which the spatial location of an object on the screen had to be remembered, and we were able to demonstrate that most of the spatial view cells encoded viewed space in allocentric (world‐based) coordinates, for their responses occurred at the same location on the large screen when it was moved to different egocentric locations relative to the macaque (Feigenbaum and Rolls 1991).

Next we recorded from these hippocampal neurons in a cue‐controlled spatial environment, and showed that most of the hippocampal neurons responded to the location in the enclosure at which the monkey was looking, relatively independently of the place where the monkey was, and we now termed these “spatial view” neurons (Rolls and O'Mara 1995).

Next we analyzed the activity of hippocampal neurons during free locomotion of the monkey walking or running with all four feet on the floor of a large laboratory providing a rich spatial environment, and showed that during free locomotion many neurons in the hippocampus *and in the parahippocampal cortex* fired to where in space the monkey was looking, and that these neurons encoded considerable information (measured using Shannon information theory) about the location in space where the monkey was looking, and little information about the place where the monkey was located, or about head direction, or about eye position (Rolls et al. 1997, 1998; Georges‐François et al. 1999).

In addition, in this test environment situation we could stop the monkey from locomoting, and were able to show that spatial view neurons fire in relation to where the macaque is looking in the room, and not to the place where the macaque is located, nor to the head direction, and nor to the eye position (see example in Figure 4) (Rolls et al. 1997, 1998; Georges‐François et al. 1999) (eye position is the term for the horizontal and vertical angles of the eye with respect to the head).

**FIGURE 4:**
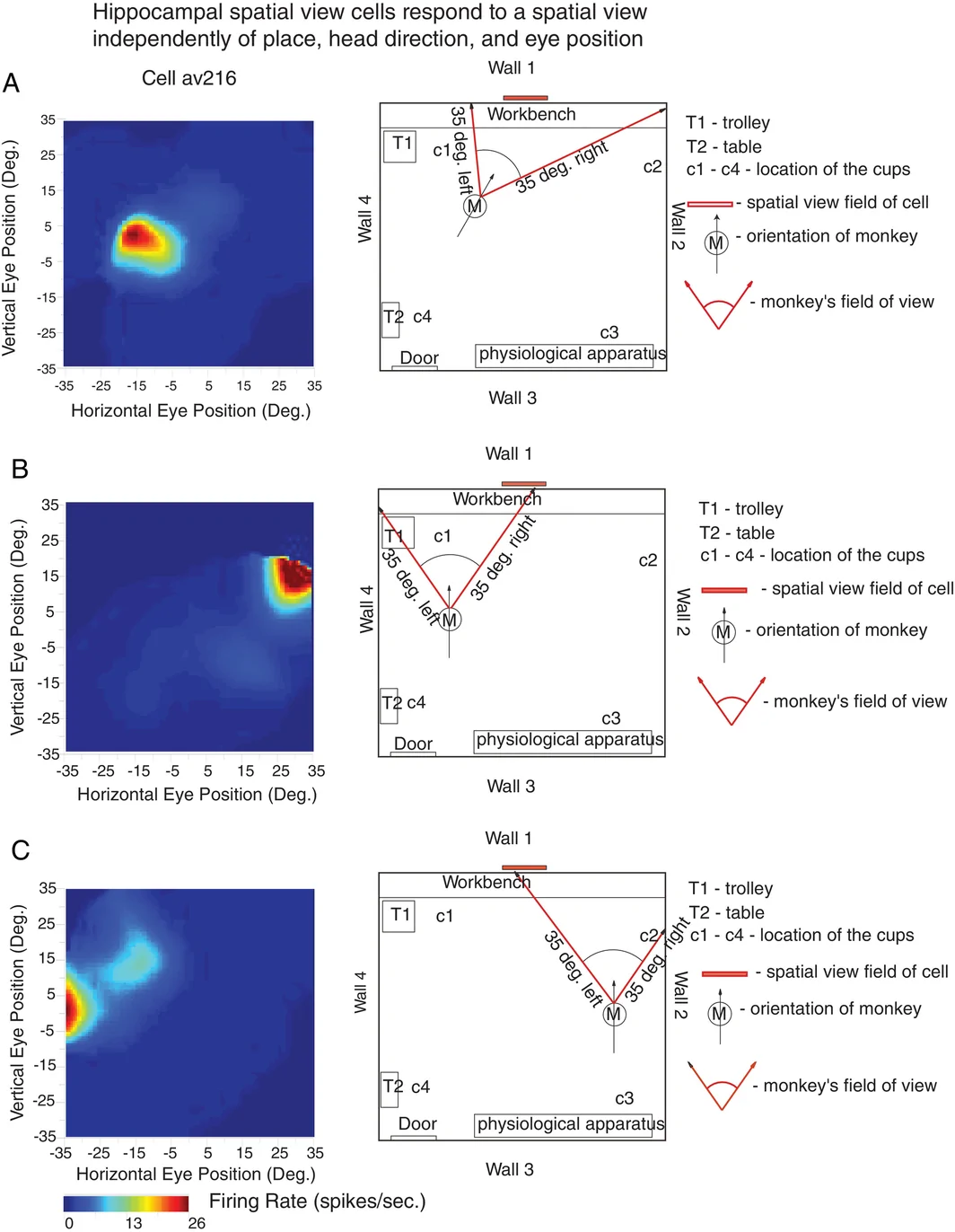
Testing of a hippocampal spatial view neuron (av216) to show that it has allocentric encoding, and that the response does not depend on where the monkey is located. The firing rate is shown as a function of the horizontal and vertical eye position, where positive values indicate right or up. The neuron responded when the monkey looked towards its view field (indicated with a red bar) relatively independently of place, eye position, or head direction. ANOVAs and information theory analyses performed on the same data cast in different ways confirmed this: For spatial view, the ANOVA was *p* < 0.001 with 0.217 bits in a 500 ms period for the average Shannon mutual information; for place *p* = 0.9 with 0.001 bits; for head direction *p* = 0.5 with 0.0 bits; and for eye position *p* = 0.8 with 0.006 bits (adapted from Georges‐François et al. 1999).

In addition, self‐motion (idiothetic) update of spatial view cells was demonstrated, in that in darkness and with black curtains obscuring any view of the lab, these (especially CA1) neurons updated their representations by firing only when the macaque moved his eyes to look towards their spatial view field (Robertson et al. 1998). Very importantly in the context of scene recall and construction, this is an example of the recall in the hippocampus of a spatial location in a scene based on self‐motion input from the eyes when the scene is not visible (Robertson et al. 1998).

More recently, evidence for neuronal spatial view representations in primates in the hippocampus and parahippocampal cortex has accumulated, including the following:

In navigation towards a goal in a virtual reality star maze with room cues, some hippocampal neurons were found to respond to where the macaque was looking (Wirth et al. 2017).

In another investigation, it was shown that many macaque neurons fire for where the monkey is facing (or, it turns out, probably was looking (Rolls 2026b)) during locomotion (Mao et al. 2021).

In another investigation, during free navigation in a 3D environment, many marmoset hippocampal neurons responded to where the marmoset was looking (Piza et al. 2024).

Further, in navigation in a virtual reality environment, some macaque hippocampal neurons were described as “landmark cells” (Vericel et al. 2024), supporting proposals and a model for how spatial view cells by coding for landmarks could be useful in navigation (Rolls 2021b).

In addition, in a virtual reality investigation involving navigation towards goals, some hippocampal neurons responded to where in space the macaque was looking, like spatial view cells, rather than the place where the monkey was located (Buffalo 2025).

Neurons with responses to viewed locations out there in space, not to the place where the individual is located, have also been found in humans (Ekstrom et al. 2003; Miller et al. 2013). For example, some medial temporal lobe neurons represented views of landmarks (Ekstrom et al. 2003). In addition, it was found that in a Treasure Hunt game, some medial temporal lobe neurons responded to the sight of remote locations rather than the individual's own place (Tsitsiklis et al. 2020). Similarly to macaque spatial view neurons, these neurons in humans respond when the spatial location is viewed with different bearings. Of especial interest, some parahippocampal cortex neurons in humans respond to scenes (Mormann et al. 2017; Mackay et al. 2024). Also, some neurons in the human parahippocampal cortex respond to the bearing and distance of viewed locations (Kunz et al. 2021). In human fMRI investigations, hippocampal activations have a bias towards the contralateral visual field (Knapen 2021; Silson et al. 2021; Groen et al. 2022; Steel et al. 2024).

## 6 How Scene Representations Are Built

An especially interesting feature of the ventromedial visual cortical scene pathway (Figure 1, green) is that it is a ventral visual cortical stream pathway for “where,” for viewed locations in allocentric space. The theory has been developed that this pathway operates computationally by forming visual feature combinations for spatial features that are close together in scenes (Rolls 2025a). These feature combination neurons are then linked using recurrent collateral connections and associative learning between co‐activated nearby feature combination neurons in a scene to form a continuous attractor model of a scene. The visual fixation scene patches are linked or stitched together across saccades, using gain modulation by gaze direction to build a representation of the whole scene (Figure 5) (Rolls 2025a). The continuous attractor networks provide for the visual features in the scene to be ordered in the correct spatial configuration with respect to each other (Rolls 2025a). The gain modulation by allocentric gaze direction allows different spatial scene patches visible in primates and humans at any one time to be linked together into a whole scene when eye saccades are made to different parts of the scene (Rolls 2025a) (Figure 5). The system is locked to allocentric space because it utilizes visual features in each scene, and their relative location forms a “chart” or “map” (Rolls and Treves 2024) of the visual scene (Rolls 2025a). This ventromedial visual “where” pathway thus produces spatial view cells that represent the location being viewed (or imagined given the idiothetic update) in space, which in the hippocampus can be associated with the object and/or reward at that location, to implement an episodic memory as described next.

**FIGURE 5:**
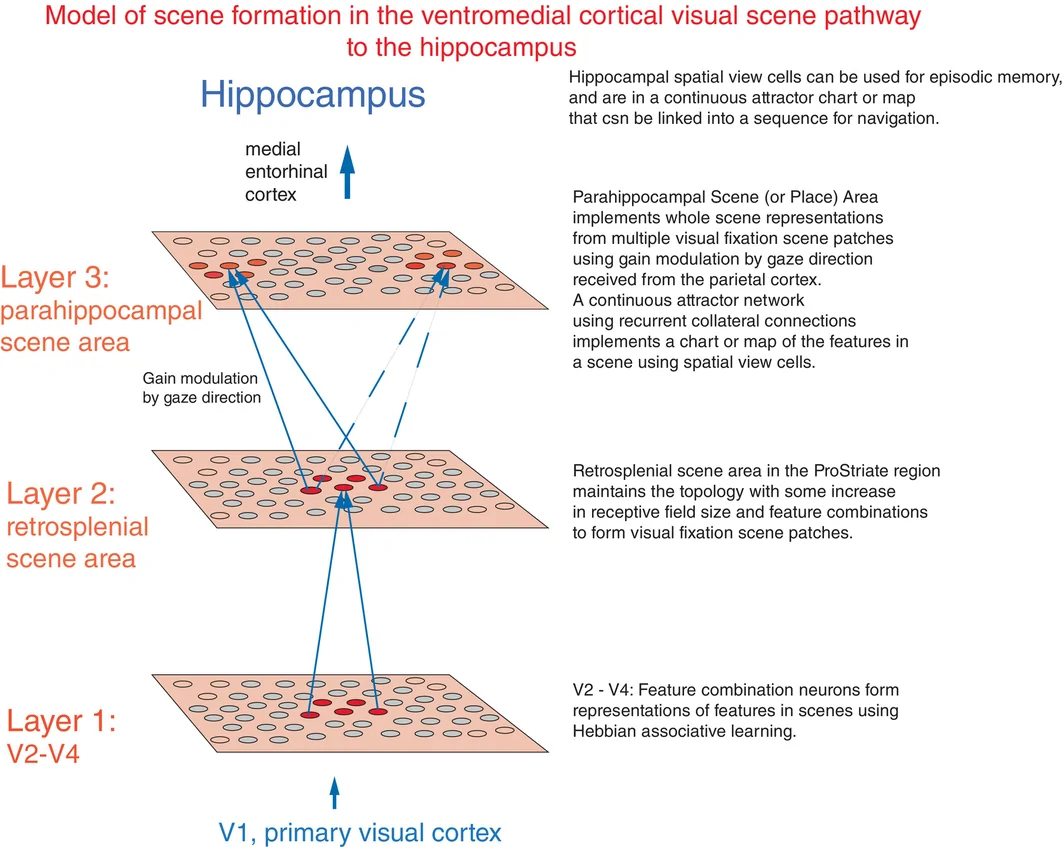
The theory and model VisSceneNet for building hippocampal spatial view cells and scene representations along the ventromedial cortical visual scene pathway. This is a three‐layer feedforward network, with competitive learning implemented using the forward synaptic connections at each layer, and short‐range convergence from layer to layer. The input is from V1 and is produced from a scene by Gabor filtering to produce a V1‐like representation. Visual fixation scene patches for a single fixation of a scene are produced by Layer 2. Layer 3 implements gain modulation by world‐based gaze direction to map visual fixation scene patches into a whole scene representation in Layer 3. The whole scene representation in Layer 3 makes use of associatively modifiable recurrent collateral connections to form a continuous attractor network for the whole scene. Nearby features in a scene are more strongly linked in the continuous attractor because they are more likely to be co‐active, but no topographical organization of space on the surface of the cortex is needed in Layer 3 (adapted from Rolls 2025a).

## 7 Combining Evidence About Scenes, Objects and Faces, and Reward Value in the Hippocampus for Episodic Memory

A theory of the hippocampus is that it can combine by autoassociation in CA3 “what” inputs about objects, faces, people etc. from the ventrolateral cortical visual stream, with “where” information from the ventromedial cortical scene stream, and with reward/affective information from the orbitofrontal cortex, to form an episodic memory (Rolls 1989a, 2026b; Treves and Rolls 1994; Kesner and Rolls 2015; Rolls and Treves 2024). Later, any one component (what, where, or reward) can trigger the retrieval in CA3 of all the components. This is an important part of the theory of scene construction described later.

Neurophysiological evidence that spatial view neurons in the primate hippocampus are likely to be involved in episodic memory includes the following. First, in an episodic memory model in which associations between “what” and “where” must be learned between what the stimulus is and where it is located in the scene, monkey hippocampal neurons take part in solving the task by learning to respond to combinations of the object shown and where it is located on a screen, for example, with responses primarily to Object 1 in spatial Location 1 on the screen (Rolls et al. 2005). Similar conjunctive or combination neurons have now been found in the human hippocampus/medial temporal lobe during a similar episodic memory task for an object and its viewed location (Kolibius et al. 2023). Supporting the importance of these neurons, damage to the hippocampal system in primates impairs the ability to remember where objects are in viewed scenes in this model of episodic memory (Gaffan and Saunders 1985; Gaffan 1994; Gaffan and Parker 1996; Murray et al. 1998).

It was also discovered that in another simple episodic memory model, hippocampal neurons in macaques respond to locations in scenes viewed on a screen where a high value fruit juice reward was obtainable if touched, while some other neurons responded to the location in the viewed scene at which a low value fruit juice reward would be obtained (Rolls and Xiang 2005). Importantly, these neurons do not code just for reward itself, for these hippocampal neurons are not engaged in an object‐with‐reward association task (Rolls and Xiang 2005), which is implemented instead by the orbitofrontal cortex (Rolls 2023d, 2026b). These hippocampal neurons can be important in scene construction by providing a reward recall cue for locations in scenes.

The operation of this system has been demonstrated in macaques in a one‐trial object‐place memory task. When the recall cue of an object that had been associated with a location in a scene was presented, then it was found that spatial view cells for the associated place were correctly recalled into activity and maintained their firing for a short period before any behavioral response could be made (Rolls and Xiang 2006) (Figure 6). This demonstrates the recall of spatial information in the hippocampus to support spatial scene processing by an object recall cue.

**FIGURE 6:**
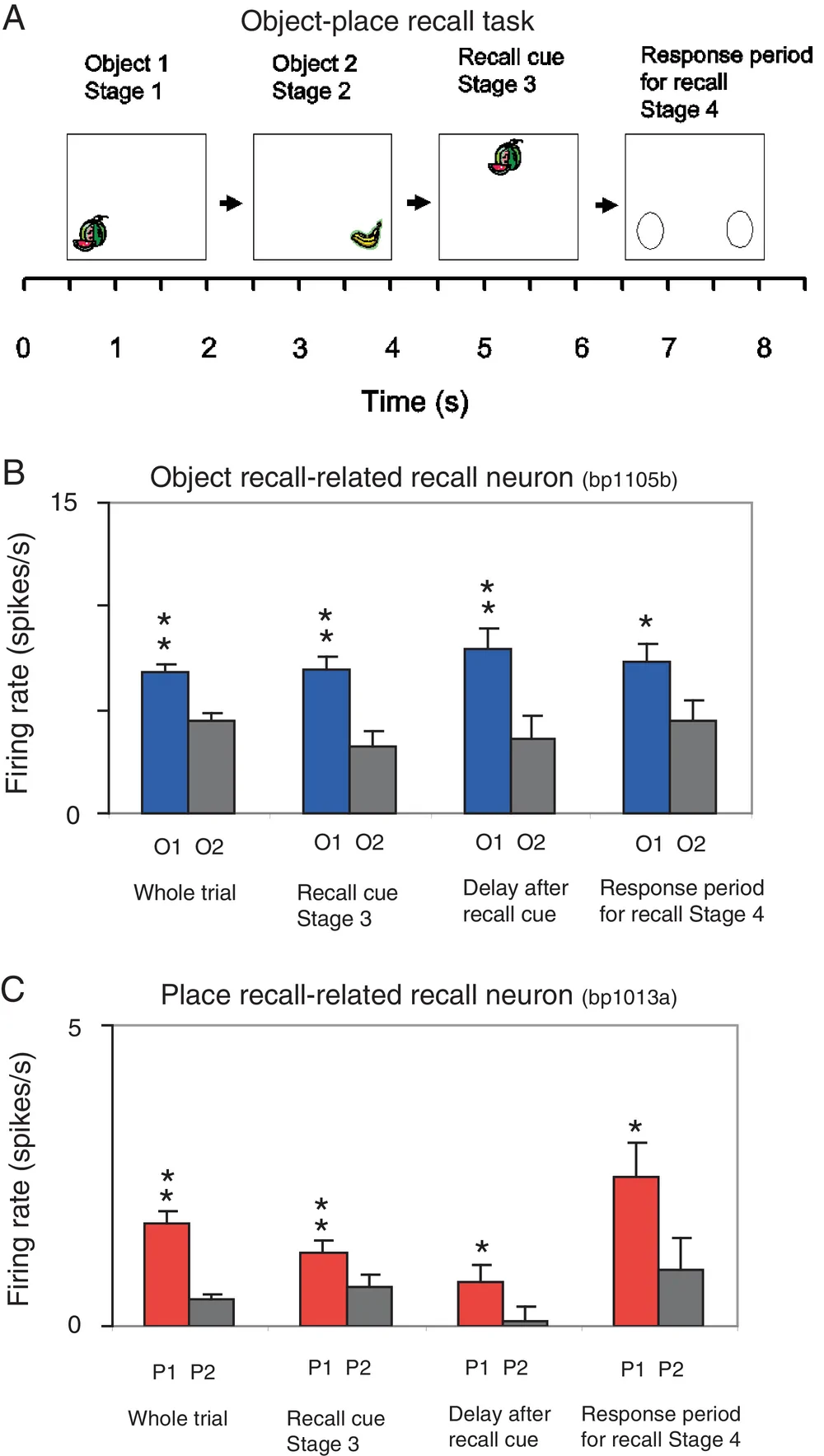
Firing of hippocampal neurons in a one trial object‐viewed location recall task. (a) In Stage 1, Object 1 was shown in a location on the screen being viewed, and in Stage 2, Object 2 was shown. In Stage 3 one of the objects was shown at the top center of the screen, and the monkey had to recall the location on the screen where that object had been shown and touch that location in order to obtain a juice reward. (b) A neuron that was selective for Object 1 (O1) responded even in Stage 4 when the object was not visible but the object and its location had to be recalled. (c) A neuron that was selective for Location 1 (P1) responded even in Stage 4 when the object was not visible but the object and its location on the screen being viewed had to be recalled. The average firing rate in spikes/s across trials ± SEM is shown. **p < 0.01; *p < 0.05. The implication is that after two objects are shown in different spatial locations, some hippocampal neurons during recall encode the location to be recalled from the object, and others encode the object to be recalled from the location (adapted from Rolls and Xiang 2006).

In humans, some medial temporal lobe neurons also reflect the learning of paired associations between views of locations and objects or people (Ison et al. 2015), or for example, between an animal and a viewed location in a scene (Kolibius et al. 2023), and more generally provide the “where” representations for episodic memory, with concept neurons providing the “what” information for episodic memory (Mackay et al. 2024), and this provides evidence that views of scenes are important for human hippocampal function.

## 8 Recalling Episodic Memories From the Hippocampus to the Neocortex

The theory of the hippocampus referred to above is the only theory that provides a quantitative account of how memories are recalled from the hippocampus back to the neocortex (Treves and Rolls 1994; Rolls and Treves 2024; Rolls 2026b, 2027). The theory of recall is that the components of the memory in CA3 are recoded by competitive learning in CA1 to make a more efficient retrieval cue. CA1 then connects via the backprojection pathways to neocortex shown in green in Figure 7 to retrieve in the backprojection connectivity via a series of pattern association networks the activity of neocortical regions for what, where, and reward/affective/value that were present during the original episode (Rolls 1989a, 2026b; Treves and Rolls 1994; Rolls and Treves 2024; Rolls, Zhang, and Feng 2024).

**FIGURE 7:**
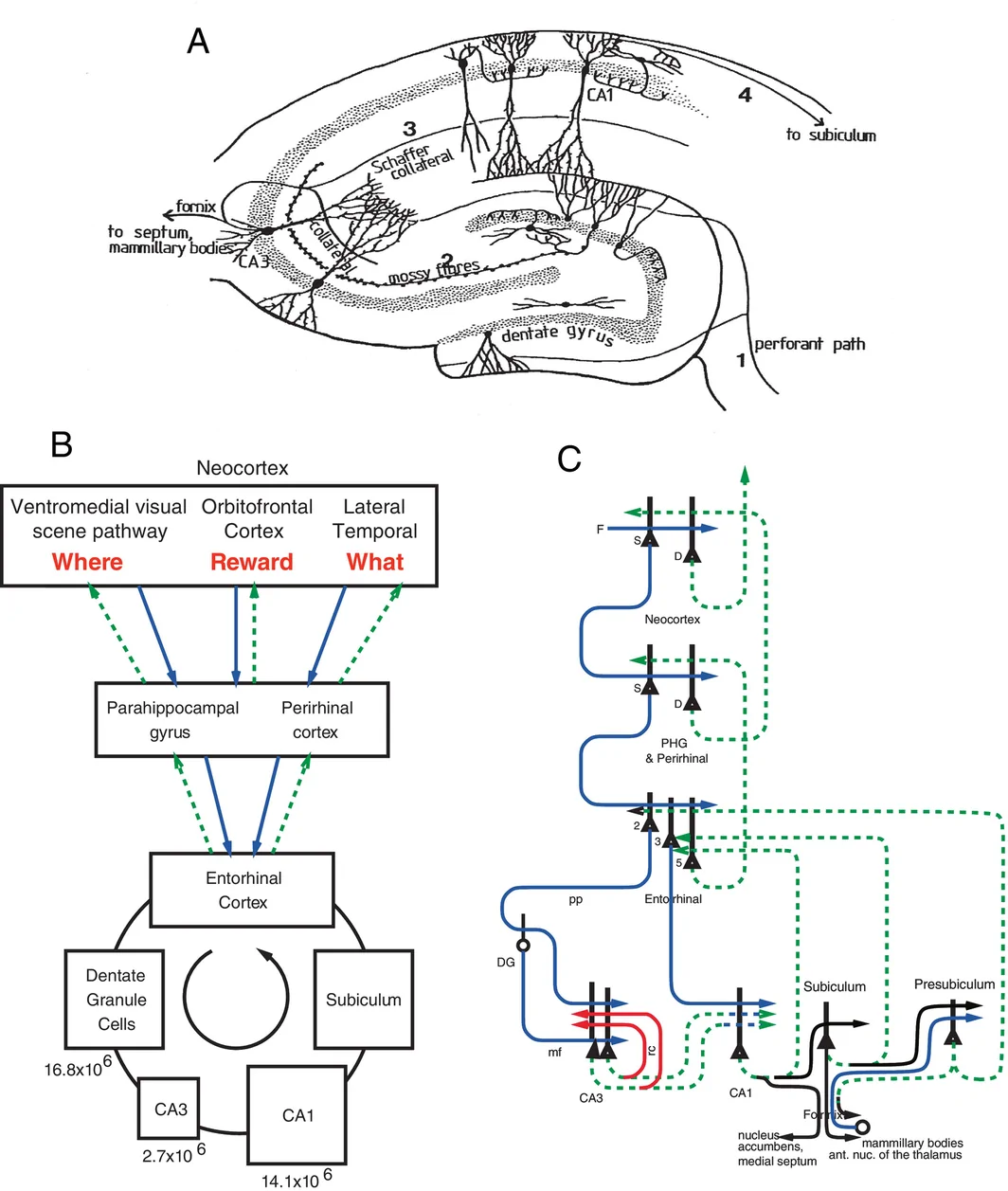
(a) Connections within the hippocampus. Inputs reach the hippocampus through the perforant path (1) which makes synapses with the dendrites of the dentate granule cells and also with the apical dendrites of the CA3 pyramidal cells. The dentate granule cells project via the mossy fibers (2) to the CA3 pyramidal cells. The well‐developed recurrent collateral system of the CA3 cells is indicated. The CA3 pyramidal cells project via the Schaffer collaterals (3) to the CA1 pyramidal cells, which in turn have connections (4) to the subiculum. (b, c). The human/primate hippocampus receives neocortical input connections (blue) not only from the “what” lateral temporal lobe and “where” ventromedial visual cortical scene pathway, but also from the “reward” prefrontal cortex areas (orbitofrontal cortex, vmPFC, and anterior cingulate cortex) for episodic memory storage; and has return backprojections (green) to the same neocortical areas for memory recall. There is great convergence via the parahippocampal gyrus, perirhinal cortex, and dentate gyrus in the forward connections down to the single network implemented in the CA3 pyramidal cells, which have a highly developed recurrent collateral system (red) to implement an attractor episodic memory by associating the what, where and reward components of an episodic memory. (b) Block diagram. (c) Some of the principal excitatory neurons and their connections in the pathways. Time and temporal order are also important in episodic memory, and may be computed in the entorhinal‐hippocampal circuitry (Rolls and Mills 2019). Abbreviations: 2, pyramidal cells in Layer 2 of the entorhinal cortex; 3, pyramidal cells in Layer 3 of the entorhinal cortex; D, deep pyramidal cells; DG, dentate Granule cells; F, forward inputs to areas of the association cortex from preceding cortical areas in the hierarchy; mf, mossy fibers; PHG, parahippocampal gyrus and perirhinal cortex; pp, perforant path; rc, recurrent collateral of the CA3 hippocampal pyramidal cells; S, superficial pyramidal cells. The thick lines above the cell bodies represent the dendrites. The numbers of neurons in different parts of the hippocampal trisynaptic circuit in humans (Rogers Flattery et al. 2020) are shown in (b), and indicate very many dentate granule cells, consistent with expansion encoding and the production of sparse uncorrelated representations prior to CA3 (Rolls 2016, 2021a).

This mechanism involved in the recall of memories from the hippocampus to the neocortex was proposed by Rolls (1989a, 1989b). The theory can be described with respect to Figure 7b,c. During episodic memory storage, neocortical inputs from different cortical regions activate the single CA3 autoassociation or attractor network, and associations between the different active CA3 neurons are learned. The CA3 neurons then drive a competitive network in CA1, and that in turn drives the backprojections back to the neocortex shown in green in Figure 7c. The active backprojection neurons with synapses onto neocortical neurons have these backprojection synapses modified by Hebbian associative learning while the neocortical neurons are still active during the storage of the episodic memory, in what is a pattern association network (Rolls 2026b). During recall, a cue is applied to one neocortical area, the CA3 attractor network performs completion, and all the backprojection synapses that were active during storage become active and recall all the neocortical components of the episodic memory by pattern association retrieval. The theory was made quantitative by Treves and Rolls (Rolls and Treves 1994; Treves and Rolls 1994). I know of no alternative theory of how the correct neurons in the neocortex are recalled by the hippocampal system backprojections to the neocortex. The theory of recall has been adopted by others (McClelland et al. 1995, 2020), though they point out that provided that the backprojection synapses are modified at some time in the way described, it would only be strictly necessary to have backprojection synapses modified at any one of the stages during the formation of the episodic memory.

## 9 Connectivity Between the Hippocampal Episodic Memory System and the Neocortical Semantic System

Episodic memory can include semantic information, for example the names, affiliations, and scientific interests of a colleague to whom one might have written to today. Given that much semantic information is stored in the neocortex including in the anterior temporal lobe (Milton et al. 2021; Rolls et al. 2022b; Rolls 2026b), this implies considerable connectivity between the anterior temporal lobe semantic system in humans and the hippocampal episodic memory system. Moreover, there is neuropsychological evidence that the hippocampal memory system may help in the formation of new semantic representations (Duff et al. 2019), and a model has been proposed for how the hippocampus by recalling related episodic memories close together in time could help learning in the anterior temporal lobe to build new semantic representations (Rolls et al. 2025). In this context, the effective connectivity between the hippocampal memory system and the neocortical semantic systems has been investigated (Rolls et al. 2025), as follows.

Figure 8 shows the effective connectivity of the human medial parahippocampal cortex regions PHA1‐3 (probably corresponding to TH in macaques, and involved in scene processing using spatial view cells) with neocortical regions (Rolls et al. 2025). This connectivity is largely with what may be termed the “Where” cortical systems concerned with representations of spatial view, and movements of objects and actions in space (Rolls 2023c, 2024, 2026b). The connectivity of the human PHA1‐3 with the hippocampus is largely via the entorhinal cortex (Figure 8) (Huang et al. 2021; Rolls et al. 2022c). First, PHA1‐3 have connectivity with the ventromedial cortical visual pathway for spatial scenes using spatial view cells (Rolls et al. 2025) including VMV1‐3, the ProStriate Cortex (ProS), and POS1 (Huang et al. 2021; Rolls et al. 2022c; Rolls 2023a, 2025a; Rolls et al. 2023a; Rolls, Yan, et al. 2024; Rolls and Turova 2025). This potentially allows scene details to be constructed from hippocampal activity during recall. Second, there is connectivity with TPOJ3, which is a semantic region in the temporo‐parietal region (Rolls 2026b). Third, there is connectivity with inferior parietal PGs (adjacent to and highly connected with PGi), which is connected with anterior temporal lobe semantic regions (Rolls et al. 2022b; Rolls et al. 2023b), and which may encode information about movements of objects, people etc. in space and actions in space (Rolls 2023c, 2026b) which is an important part of what may need to be reconstructed when a scene is recalled (Rolls et al. 2022b; Rolls 2023c; Rolls et al. 2023b; Rolls, Feng, and Zhang 2024). These visual inferior parietal cortex regions, PGi and PGs, have connectivity for semantic representations of this type, because these regions are at the top of the visual parietal cortex hierarchy involved in spatial computations for actions (Rolls et al. 2023b), but also have connectivity with anterior temporal lobe regions with semantic representations of objects and people, and with superior temporal sulcus (STS) regions involved in social interactions (Rolls et al. 2022b; Rolls et al. 2023b; Rolls 2026b). Fourth, there is connectivity from some area 7 parietal and related regions involved in visuo‐motor actions in space (7Pm, 7Am, 7PL, IP0 and PCV) (Rolls, Deco, et al. 2023). Fifth, there is connectivity with reward‐related ventromedial prefrontal cortex region 10r (Rolls 2022, 2023b; Rolls et al. 2022a).

**FIGURE 8:**
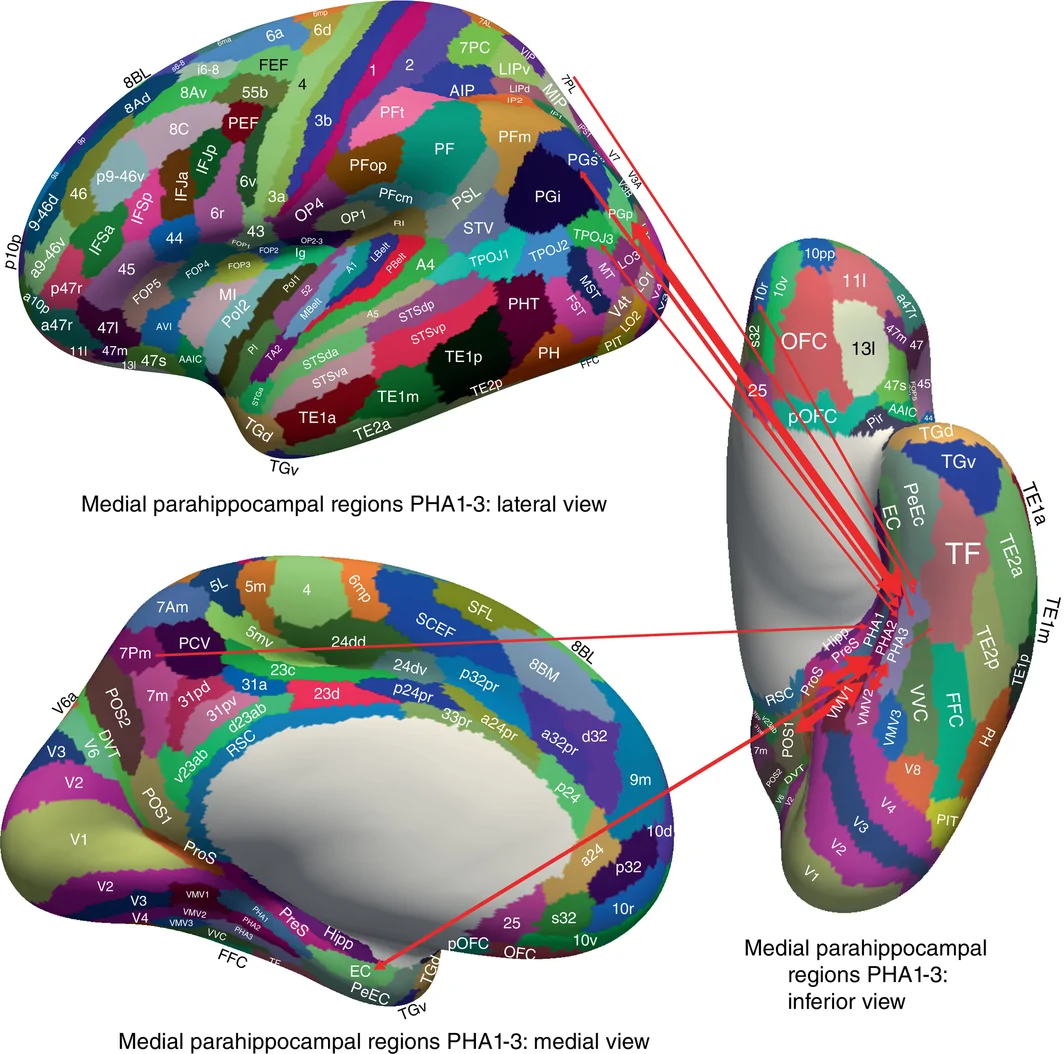
Connectivity of the hippocampal memory system includes connectivity via the medial parahippocampal cortex PHA1‐3 not only with the ventromedial scene pathway including VMV regions, but also with the parietal cortex including inferior parietal cortex visual regions PGs and PGp. The effective connectivity between cortical regions in the Human Connectome Project Multimodal Parcellation atlas (HCP‐MMP) (Glasser, Coalson, et al. 2016; Huang et al. 2022) is shown. A list of the cortical regions in the HCP‐MMP atlas is shown in Supporting Information. The width of the arrows represents the magnitude of the effective connectivity, and the size of the arrowheads the magnitude in each direction. The data are from effective connectivity matrices computed previously from resting state timeseries in 171 HCP participants imaged at 7T (Rolls et al. 2022b, 2022c; Rolls et al. 2023a) (adapted from Rolls et al. 2025).

Figure 9 shows connectivity via the human lateral parahippocampal cortex area TF to anterior temporal lobe memory systems (Rolls et al. 2025) that represent objects and people (Rolls 2023c, 2026b), which may be important when some scenes are reconstructed from memory, or using prospective thinking. When an episodic memory is stored, this connectivity enables semantic representations of objects and people represented, for example, by concept cells (Quian Quiroga 2026) to be part of an episodic memory, as described in Section 10. When a whole memory is reconstructed, this allows semantic representations in these neocortical regions of objects and people, and so forth, to be brought into activity, as described in Section 10.2.

**FIGURE 9:**
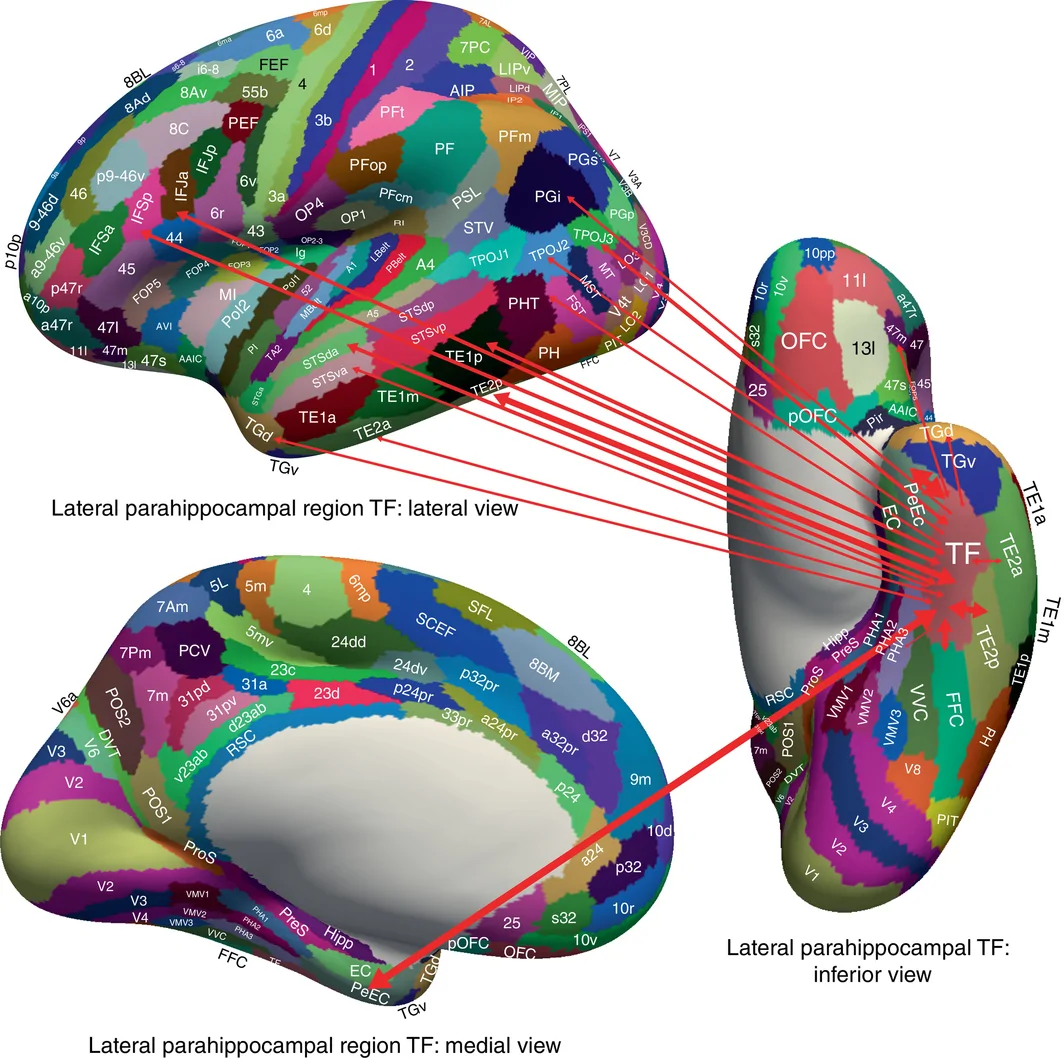
Connectivity of the hippocampal episodic memory system includes connectivity with many cortical regions in and connected to anterior temporal lobe semantic memory systems. The connectivity of the lateral parahippocampal cortex region TF, which includes connectivity to semantic systems related to “What” representations, is shown (see text). The effective connectivity between cortical regions in the Human Connectome Project Multimodal Parcellation atlas (HCP‐MMP) (Glasser, Coalson, et al. 2016; Huang et al. 2022) is shown. A list of the cortical regions in the HCP‐MMP atlas is shown in Supporting Information. The width of the arrows represents the magnitude of the effective connectivity, and the size of the arrowheads represents the magnitude in each direction. The data are from effective connectivity matrices computed from resting state timeseries in 171 HCP participants imaged at 7T (Rolls et al. 2022b, 2022c; Rolls et al. 2023a) (adapted from Rolls et al. 2025).

## 10 A Theory of Mental Scene and Memory Construction

### 10.1 Mental Scene Construction

We now have key elements in place to describe a neurocomputational theory of scene and memory construction.

Part of the process of scene construction for at least recent memories is like the recall of an episodic memory. Perhaps one receives an external memory retrieval cue, such as seeing a friend (a “what” recall cue), which leads to recall of where one saw the friend most recently, and indeed of the whole scene in which that autobiographical event occurred. One might remember later the whole scene in which one last saw the friend, such as the lecture theater in which the friend was giving the lecture, where the friend sat before and after the lecture, as well as what the friend lectured about, and what he or she was wearing.

The theory proposed here by which this type of “scene construction” or scene recall occurs is that the inferior temporal cortex as part of the visual “what” system would activate the neurons that represent the sight of that person, and this would project via the perirhinal cortex and entorhinal cortex to the hippocampus, and in CA3 the whole memory would be recalled by autoassociation recall of where one saw the person, and also of the reward or affective components (see Figure 7 for the architecture; Rolls and Treves 2024; Rolls 2026b). The CA3 would then activate CA1, which would start the recall process back to the neocortex via the entorhinal cortex (Rolls and Treves 2024; Rolls 2026b), which would activate the scene representation in the medial parahippocampal cortex (regions PHA1‐3). This memory retrieval process with a retrieval cue from one part of the neocortex, for example, “what” from the inferior temporal visual cortex (Rolls 2026b, 2026c), or, for example, “reward” from the orbitofrontal cortex, to retrieve the other parts of the whole memory back in, for example, the “where” neocortex including the medial parahippocampal cortex PHA1‐3, VMV1‐3, and so forth (Figure 8), has been validated at the integrate‐and‐fire neuron network model level (Rolls, Zhang, and Feng 2024). The same would apply to the recall of the affective components of the episodic memory in the orbitofrontal cortex.

The representation of the recalled, constructed scene would be as described in Section 6, namely for a part of the scene about the size of the spatial view field of a medial parahippocampal cortex neuron. The scene representation in the medial parahippocampal cortex (the parahippocampal scene or place area) would be that of a continuous attractor network in which the neurons are the spatial view cells, as indicated in Figure 5 (Rolls 2025a). The continuous attractor representation of the scene ensures spatial continuity across the scene, with each part of the scene therefore ordered correctly with respect to other parts of the scene because of the synaptic associations learned for nearby locations in the scene (Rolls 2023c, 2026b; Rolls and Treves 2024). The part of the scene being imagined at any one time, which as noted above might be about the size of a hippocampal or parahippocampal cortex spatial view field, would then be shifted to another part of the scene by the inferior parietal idiothetic signal from inferior parietal cortex region PGp using gain modulation as described and modeled for VisSceneNet (Rolls 2025a). Because this idiothetic update mechanism for position in a scene normally involves eye movements (Rolls 2025a), this mechanism of internally viewing different parts of a scene by using eye movements might also occur during scene construction, as the mental image moves from one part of a scene to another. I propose that this is why eye movements often occur during mental scene construction (Setton et al. 2024).

Another type of recall cue for a spatial scene memory might be a reward or punishment‐related stimulus (with the pathway from the orbitofrontal cortex to the hippocampus shown in Figure 1), such as an extra‐large wave coming towards us when we are windsurfing, which can be punishing, or rewarding if we ride it well. That might trigger a memory of what happened on a previous weekend, when a mast‐high wave was barrelling towards us, which was definitely potentially punishing, but which we managed to outrun and sail off to the side where the wave was not barrelling or breaking (which was rewarding). The recall of such an event can produce a complete spatial scene recall, of exactly where one was in Kimmeridge Bay on the South Coast of England, of the exact scene in front of one with a rocky shore ahead and a green hill behind, and with the safe landing ramp on one's right. The computational steps involved in this recall of the scene might start in the orbitofrontal cortex with the decoding of the reward/punishment value of the wave being seen now or remembered, which would activate the hippocampus via the ventromedial prefrontal cortex and parts of the pregenual anterior cingulate cortex reward/affective system (Section 4 and Figure 1), and then via the perirhinal and then entorhinal cortex, and in part via the posterior cingulate cortex (with both paths shown in Figure 3). In hippocampal CA3 the reward/punishment input would trigger recall of the rest of the whole memory (i.e., the scene and the “what” part). That would then via CA1 recall the scene component back to the medial parahippocampal scene “where” cortex, as shown in Figure 7 and described fully elsewhere (Treves and Rolls 1994; Rolls and Treves 2024; Rolls, Zhang, and Feng 2024; Rolls 2026b). The ventromedial prefrontal cortex (vmPFC) is part of this route, and does have very pronounced connectivity with the hippocampal memory system, in both directions, though stronger towards the hippocampus (Figure 3) (Rolls 2022; Rolls et al. 2022a) (the return direction is for the recall of the emotional/reward component of a memory from the hippocampus back to the orbitofrontal cortex in response to a “what” or “where” recall cue).

The ventromedial prefrontal cortex/pregenual anterior cingulate cortex region has been implicated in scene construction by fMRI activation and brain damage investigations (Maguire and Mullally 2013; Bonnici and Maguire 2018; McCormick et al. 2018, 2020; Ciaramelli et al. 2019) (but see De Luca et al. 2019). The reward cue‐initiated scene construction described in the preceding paragraph provides an approach to understanding the roles of the vmPFC in scene construction. The vmPFC is a key region on the pathway from the orbitofrontal cortex, where rewards, punishers, and emotions are represented (Rolls 2019, 2023d, 2026b), to the hippocampus (via the perirhinal and entorhinal cortex, and via the posterior cingulate cortex), as shown in Figure 3. This is supported not only by fMRI effective and functional connectivity and diffusion tractography in humans (Rolls et al. 2022a) but also by tract‐tracing in macaques, which reveals direct connections from CA1 to the vmPFC/anterior cingulate cortex, as well as connections from the vmPFC to the hippocampal system via perirhinal and entorhinal cortex (Bunce et al. 2013; Wang et al. 2021).

What is therefore proposed, based on this evidence and that in the preceding paragraphs in this section, is that the roles of the vmPFC in scene construction are to provide the reward‐based input evidence to the hippocampal memory system that is used to initiate memory recall, of in this case the scene components (e.g., the scene where one last heard a very good lecture). The implication is that the vmPFC is not involved in scene processing itself, but has the role of providing a recall cue to the hippocampal memory system for scene construction. Indeed, given the connectivity just described, the hypothesis is that the reward information reaches the hippocampus by its non‐spatial input routes (the perirhinal cortex and not the lateral parahippocampal cortex), that the hippocampus then retrieves the whole memory including the spatial component, and then via the backprojections to the medial parahippocampal cortex PHA1‐3 produces recall of the whole scene. This hypothesis is supported by the fact that the medial parahippocampal cortex receives spatial scene inputs from the preceding ventromedial visual (VMV) regions which in turn receive from the ProStriate cortex all of which are involved in scene processing as described in Sections 3 and 4, whereas the vmPFC does not receive these spatial inputs, except as part of a recall operation mediated by memory recall in the hippocampus. This hypothesis is also supported by the finding that lesions of the vmPFC/anterior cingulate cortex in humans do not impair scene processing per se, in that the individuals are not impaired in scene processing tasks that require detecting for example constructive scene violations such as an endless staircase (Ciaramelli et al. 2019; De Luca et al. 2019). This hypothesis is also supported by the finding that during the recall of autobiographical memories, activations in the vmPFC reflect the reward and affective value of the memories being recalled (Lin et al. 2016). Also, given the route from the vmPFC via the posterior cingulate cortex to the hippocampal memory system (Figure 3) (Rolls et al. 2022a), the present hypothesis can account for activations of the posterior cingulate cortex (the postero‐ventral memory‐related part; Rolls, Wirth, et al. 2023) that occur during the recall of autobiographical memories (Maddock et al. 2001; Markowitsch and Staniloiu 2023).

A further issue is that although fMRI investigations may show some signatures of scene processing in the vmPFC, this may not be surprising, given the strong connectivity just referred to between the hippocampus and the vmPFC (Rolls et al. 2022a).

An implication of the approach taken here is that scene representations per se are built through the ventromedial visual cortical scene pathway to the medial parahippocampal cortex (Rolls 2025a) (Figure 5), and are then used in the hippocampus as the “where” part of an episodic memory with “where,” “what,” and reward components (Rolls and Treves 2024; Rolls 2026b). Consistent with this, Larry Squire and colleagues have evidence that hippocampal damage in humans impairs episodic memory but not spatial processing involved in, for example, navigation and scene analysis, which may be related more to damage to the parahippocampal cortex (Rungratsameetaweemana et al. 2019; Urgolites et al. 2019).

Although much of the hippocampus, parahippocampal scene area, and retrosplenial scene area cortex are activated when viewing scenes, the anterior parts of these regions show a bias to relatively more activity during scene recall (“/reconstruction”) (Section 2; Zeidman et al. 2015; Zeidman and Maguire 2016; Steel et al. 2021)). Why might this be? The computational neuroscience hypothesis that I propose is that the anterior parts of the hippocampus (and thereby these other regions) are more likely to be activated from the perirhinal cortex and lateral/anterior entorhinal cortex, which is how the “what” and reward inputs used to cue scene retrieval enter the hippocampus. In contrast, when the activation is produced by actually viewing a scene, the main input to the hippocampus will come from the medial parahippocampal cortex PHA1‐3, which in humans tends to be posterior to the perirhinal cortex (see Figure 1 and Figure S1).

### 10.2 Mental Whole Memory Construction and Prospective Thinking

When scenes are constructed, people and objects and rewarding stimuli may be present in the spatial scenes being recalled, or imagined in prospective thinking. The hippocampus is ideally placed to implement this aspect of scene and memory construction because it has connectivity via the lateral parahippocampal cortex TF to the anterior temporal lobe (Figure 9) where semantic representations of objects and people appear to be stored (Rolls 2023c, 2026b). Similarly, reward components of a spatial scene can be recalled or imagined via the connectivity from the hippocampus via entorhinal and perirhinal cortex to the orbitofrontal cortex (Figure 1).

The highly developed connectivity between the hippocampal system shown in Figure 9 between the lateral parahippocampal gyrus (TF) and the semantic systems in the anterior temporal lobe such as TGv, TE2a, and temporo‐parietal junction (TPOJ1‐3) (Rolls et al. 2022c, 2025) emphasizes that semantic concepts such as a particular person with all their attributes (such as face appearance, sound of voice, written name, heard name, friends and colleagues, etc.) are in humans not only what can be included in an episodic memory, but can also be recalled from the hippocampus. This does not mean that all the attributes have to be independently stored in the hippocampus. Instead, given that “concept cells” are found in the anterior temporal lobe up to and including the temporal pole (Dornier et al. 2025), as well as more posteriorly in the medial temporal lobe (Quian Quiroga 2026), concept cells provide an appropriate semantic representation as an input to the hippocampal episodic memory system. This is because each concept cell can respond to many of the different semantic attributes of a person, object, country, and so forth (Quian Quiroga et al. 2005; Quian Quiroga 2012, 2026; Rutishauser et al. 2021). Neurons with multimodal representations of stimuli that may be non‐linguistic precursors of concept cells are starting to be discovered in non‐human primates (Rolls 2023b).

I emphasize that when an episode memory is retrieved, then semantic components will be retrieved into activity in the anterior temporal lobe, with the same concept cells that were active during episodic memory storage being retrieved by the mechanism using the backprojection pathways from the hippocampal system acting by pattern association recall, as described in Section 8 (Treves and Rolls 1994; Rolls and Treves 2024). The next process that occurs allows whole memories including semantic components to be constructed, I now propose. The process is that once a memory has been recalled from the hippocampus to the neocortex (including semantic regions), whatever neocortical representations are now being activated then act as a further memory retrieval cue, in which the now more complete neocortical representation acts as a new recall cue to the hippocampus, which with this more complete retrieval cue of the memory acts on the hippocampal system in CA3 to perform a second iteration of retrieval within the hippocampus, which then retrieves a more complete memory in the neocortex. This “long‐loop” attractor (Rolls 2026b) involving neocortex to hippocampus to neocortex repeated iterations was highly evident in our integrate‐and‐fire simulation of the neocortical‐hippocampal‐neocortical system, in which each “iteration” round the whole loop took 100–200 ms (Rolls, Zhang, and Feng 2024) (with the architecture for the simulation shown in Figure S2). This progressive recall involving this long loop neocortex to hippocampus to neocortex attractor (with more attractor dynamics added in the CA3) is proposed to lead to a construction of a whole memory that gradually develops over a few seconds.

The iterated recall of a whole memory may be subject to noise caused by the random spiking times of neurons for a given mean firing rate, as explained elsewhere (Rolls and Deco 2010; Rolls 2026b), and that noise may make the neocortical local attractor network jump to a closely related semantic representation, as in my theories of creativity and of dream sleep (Rolls 2026b). That may enable a train of thought through closely related episodic memories with similar semantic content, producing a train of remembered thoughts. And these remembered thoughts may in turn produce jumps to further nearby locations in semantic/episodic memory space, leading to a whole train of thought constructed in this way, and moving to new places in semantic space, thus providing an account of constructed prospective thought and memory. I propose that the hippocampus may be useful in these iterative long loops because these long loops allow different semantic associations that develop through several seconds of recall time in the neocortex to find further semantic associates that may have been present in related episodic memories.

An implication of this iterative process of neocortical‐hippocampal‐neocortical recall is that each time that we retrieve an episodic memory, this recall‐related iterative processing may result in the recalled representations changing somewhat because of the iterative constructive process between the neocortex and hippocampus, as just described. This iterative processing may contribute to what we think of as episodic memories being stored again in somewhat altered form, which may I propose contribute to the recall of episodic memories that occurs close to the original event being more close in content to the original event, with some development and change of what is remembered by further memory storage each time that the memory it recalled and iteratively processed back to neocortex further. This is relevant to memory processes such as eye‐witness testimony.

These concepts also provide an approach to understanding the relation between hippocampal episodic memory and autobiographical memory, which is a long‐term type of neocortical memory that, after storage, remains after hippocampal damage. I point out that human episodic memory is a “first person” type of memory: it is a memory of what happened to us at a particular time and in a particular place. Even if we learned a fact during the episode, the episodic memory is still a memory of the time and place when and where we learned that fact. The sequitur of this point is that the processes just described of hippocampal‐neocortical interactions for storage and recall are ideally suited for setting up autobiographical memory as a set of episodic memories retrieved from the hippocampus that are stored in the neocortex in a long‐term form that are autobiographical memories. The approach described here thus provides a computational approach to understanding how autobiographical memories are constructed from episodic memories, which are “first‐person.”

What I have just proposed is not then just a mechanism for the construction of a whole memory with semantic content, but it also provides a computational approach to how prospective thinking and memory construction could be helped by the hippocampal episodic memory system iteratively interacting with the neocortical semantic system. The proposal of mental memory construction also provides an account of the relation between hippocampal episodic memory, which I point out is “first‐person,” and long‐term autobiographical memory which is by definition “first‐person.”

## 11 Discussion

A theory of how scene representations are constructed in response to a retrieval cue such as an object or person, or a signal of reward or affective value (such as the sight, smell and taste of a madeleine; Proust 1913) from the orbitofrontal cortex, has been described in this paper. The theory shows how a representation of a scene is encoded by spatial view cells in the medial parahippocampal cortex, and how that spatial representation is recalled using the hippocampal memory system via backprojections from the hippocampus (via the entorhinal cortex) to the medial parahippocampal cortex. The hippocampus itself recalls in CA3 the scene associated with the object or reward retrieval cue. The theory also shows how different parts of a scene are stitched together in the correct spatial configuration in a continuous attractor network in the medial parahippocampal cortex (and perhaps hippocampal CA3); and how eye movements may occur when different parts of the whole scene are inspected mentally because of the way in which idiothetic update of the location being looked at in a scene can normally be performed using gain modulation by eye gaze direction. The theory also is that the vmPFC is not a spatial computer itself like the medial parahippocampal cortex/hippocampal system, but is related to scene construction because the vmPFC provides one of the key inputs to the hippocampus that drives scene reconstruction, namely the reward or affective value of a stimulus that leads to the recall of the spatial scene. Considerable evidence from neuropsychology was described that is consistent with this interpretation of the role of the vmPFC in providing a recall cue to the hippocampus episodic memory system, rather than of performing spatial computation.

This theory applies naturally when a scene is constructed by the hippocampus in response to an object or affective recall cue for an episodic memory. Moreover, it is not surprising if the vmPFC is activated during autobiographical memory recall, because most autobiographical memories have a reward, punishment, or emotional component, which is likely to trigger many autobiographical memories. The theory also may be applied to prospective thinking, for during that process objects or rewards may need to be associated with spatial locations in scenes as part of a plan, and the parts of that plan may engage the hippocampal episodic memory system so that the associates that may be part of a plan can be remembered.

What about the construction of scenes related to autobiographical memory? The hippocampus is implicated in autobiographical memory recall in that a wealth of neuropsychological studies complement the fMRI findings of hippocampal activation during autobiographical memory recall by showing that patients with bilateral hippocampal damage are either unable to recall autobiographical memories at all or that retrieval is significantly impoverished (see McCormick et al. 2020). If the autobiographical memory is for a relatively recent event, the hippocampal memory system may be involved in scene construction in the way just described for episodic memory. But if the autobiographical memory is for an event in the remote past, the situation may be more complicated, because the memory capacity of the hippocampus is limited, and memories may need to be retrieved to the neocortex from the hippocampus for long‐term storage and consolidation in the neocortex (Rolls 2022, 2026b). In more detail, hippocampal theory is that the hippocampal episodic memory system has a limited capacity in the order of 17,500 memories (given the 17,500 recurrent collateral synapses on each CA3 cell; Watson et al. 2025; Rolls and Treves 2024; Rolls 2026b). In this situation, is remote autobiographical memory stored outside the hippocampus, perhaps in the anterior temporal lobe? In Section 10.2 a theory was described of how “first‐person” episodic memories could be key to understanding how autobiographical memories are formed, and a mechanism was proposed. In Section 10.2 the scene construction theory was extended to provide a computational framework for understanding how iterative processing in a neocortical‐hippocampal‐neocortical loop that is very evident in simulations of the hippocampal system (Rolls, Zhang, and Feng 2024) could enable the construction over a few seconds of whole mental including semantic components of a memory. Section 10.2 also considers how semantic content can be part of a hippocampal episodic memory.

The research described here is compared with some previous research next. A mechanism for the retrieval of object locations in terms of the mental imagery of an array of objects being visualized in the classic Milan square neuropsychological test (Bisiach and Luzzatti 1978) has been described (Byrne et al. 2007; Bicanski and Burgess 2018). The model is based on the place, head‐direction, and grid cells, allocentric boundary‐cells, and object‐vector cells found in rodents, and so does not model the systems and pathways found in primates including humans for mental imagery and memory reconstruction that utilize spatial view cells to encode scenes in the ventromedial visual cortical stream, visual object and face cells in the ventrolateral visual cortical stream, or for that matter reward cells found in the orbitofrontal cortex (Rolls 2026a, 2026b, 2027). Nor is there any quantitative theory in that model (Byrne et al. 2007; Bicanski and Burgess 2018) of how the hippocampus is able to recall the correct neocortical neurons into activity during mental scene and memory construction from an episodic memory (Treves and Rolls 1994; Rolls and Treves 2024; Rolls 2026b). That model (Byrne et al. 2007; Bicanski and Burgess 2018) also considers transforms between egocentric and allocentric coordinate frames, though how these coordinate transforms occur in the primate including human visual system is now thought to take place in a series of stages from retinal to head‐based coordinates, and then to allocentric bearings to a landmark, and then to allocentric spatial view coordinates along the dorsal visual stream to the parietal cortex (Rolls 2020). The encoding and retrieving of the location of arrays of objects in a scene has also been modeled based on hippocampal place cells and entorhinal cortex grid cells as follows: (Hasselmo 2009). In the model associations were formed between a place cell vector p(t) encoding a trajectory and a vector o(t) representing the stimulus features of individual objects or events experienced at specific positions on a trajectory. The object vector was active when the agent was within a specific distance of the object location (see also Hasselmo et al. 2010; Robinson et al. 2026). The neural activity associated with coding of place along a trajectory that may be useful in path integration has also been analyzed using fMRI in humans (Chrastil et al. 2015, 2016).

In conclusion, this paper presents a neurocomputational theory of mental scene construction, and further the mental construction of whole memories, during memory recall, prospective thinking, and autobiographical memory. The theory describes the nature of the representation of scenes using spatial view cells in a continuous attractor network in the medial parahippocampal cortex; the ventromedial visual cortical pathways and how scene representations in the medial parahippocampal cortex are built along this pathway; how scene representations can be recalled in the medial parahippocampal cortex via hippocampal memory completion in response to an object or reward value/affective recall cue; the role of the vmPFC in these processes in the reward/affective processing but not in spatial computations; semantic contents in episodic memories; the construction of whole mental memories; and the relation between “first‐person” hippocampal episodic memory which may be autobiographical, and long‐term neocortical autobiographical memory.

The author does not know of any other theory of or approach to scene construction that combines neurophysiological evidence about how scenes are represented in primates including humans by spatial view cells (Georges‐François et al. 1999; Rolls 2025b, 2026c); details of the effective connectivity of the what, where and reward pathways to and from the hippocampus in humans (Rolls et al. 2022a, 2022c; Rolls 2023d, 2024; Rolls et al. 2023a); evidence from neurophysiology and fMRI about the reward as well as spatial and object systems in the primate including human brain (Grabenhorst and Rolls 2011; Rolls 2023d, 2025b, 2026b; Rolls, Feng, and Zhang 2024; Rolls, Zhang, et al. 2024; Zhang et al. 2026a); a quantitative computational theory of how the hippocampus can store memories and then recall them to neocortex (Rolls and Treves 2024; Rolls, Zhang, and Feng 2024), and of how scenes representations are constructed (Rolls 2025a); and evidence from human neuropsychology. The author also does not know of any other theory or approach that takes all of the above, and combines this with the connectivity of the hippocampal system with semantic systems utilizing concept cells (Quian Quiroga 2026) in the human anterior temporal lobe (Rolls et al. 2022b) and inferior parietal cortex (Rolls 2023c; Rolls et al. 2023b) with integrate‐and‐fire network simulations of the hippocampus‐neocortical circuitry (Rolls, Zhang, and Feng 2024) that emphasize progressive recall of whole memories for mental memory construction by using iterative interactions between these neocortical semantic and spatial regions and the hippocampus.

## Author Contributions

The author wrote the paper.

## Funding

The author has nothing to report.

## Disclosure

The author has nothing to report.

## Ethics Statement

No data were collected as part of the research described here.

## Conflicts of Interest

The author declares no conflicts of interest.

## Supporting information


**Table S1:** Regions defined in the modified Human Connectome Project atlas (Glasser et al. 2016). L = left hemisphere, R = right. The column ‘Reordered region ID’ is that used in figures 1–5, and is a reordering of that based on suggestions in the supplementary information of Glasser et al. (2016). In that supplementary information of that paper, the 360 regions are grouped based on geographic proximity and functional similarities, which was reorganized and provided by Dr. Dianne Patterson of the University of Arizona at https://neuroimaging‐core‐docs.readthedocs.io/en/latest/pages/atlases.html with the HCP‐MMP_UniqueRegionList.csv and is shown in the column labeled CortexID in Table S1. The volumes are in mm^3^. This modified atlas with the reordering is described elsewhere (Huang et al. 2022).
**Figure S1:‐1:** Example coronal slices showing regions defined in the HCPex atlas and added subcortical regions (Huang et al. 2022). The abbreviations are as in Table S1. The *y* values for the coronal slices are in MNI coordinates.
**Figure S1:‐2:** Example coronal slices showing regions defined in the HCPex atlas and added subcortical regions (Huang et al. 2022). The abbreviations are as in Table S1. The *y* values for the coronal slices are in MNI coordinates.
**Figure S1:‐3:** Example coronal slices showing regions defined in the HCPex atlas and added subcortical regions (Huang et al. 2022). The abbreviations are as in Table S1. The *y* values for the coronal slices are in MNI coordinates.
**Figure S1:‐4:** Example coronal slices showing regions defined in the HCPex atlas and added subcortical regions (Huang et al. 2022). The abbreviations are as in Table S1. The *y* values for the coronal slices are in MNI coordinates. (b) Anatomical regions of the human visual and other cortical regions. Regions are shown as defined in the HCP‐MMP atlas (Glasser et al. 2016), and in its extended version HCPex (Huang et al. 2022). The regions are shown on images of the human brain without the sulci expanded to show which cortical HCP‐MMP regions are normally visible, for comparison with figures 6–10 (the ICBM153 MNI T1 image was used to prepare this figure). Abbreviations are provided in Table S1.
**Figure S1:‐5:** Anatomical regions of the human visual and other cortical regions. Regions are shown as defined in the HCP‐MMP atlas (Glasser et al. 2016), and in its extended version HCPex (Huang et al. 2022). The regions are shown on images of the human brain without the sulci expanded to show which cortical HCP‐MMP regions are normally visible (the ICBM153 MNI T1 image was used to prepare this figure). Abbreviations are provided in Table S1.
**Figure S1:‐6:** Regions in the Human Connectome Project Multimodal Parcellation atlas (HCP‐MMP) (Glasser et al. 2016) and its extended version HCPex (Huang et al. 2022) to show the cortical regions. The regions are shown on images of the human brain with the sulci expanded sufficiently to allow the regions within the sulci to be shown. Abbreviations are provided in Table S1. For comparison, a version of this diagram without the sulci expanded is provided in Figure S1‐5.
**Figure S2:** Simulation of neocortical ‘what’ and ‘where’ inputs to the hippocampus for the storage of episodic memory, and for the recall of ‘what’ (object or face) and ‘where’ (spatial view) information back to the ‘what’ and ‘where’ neocortex. The pyramidal cell bodies are shown as triangles, the dendrites as the thick lines above the cell bodies, and the axons as thin lines terminated with an arrow. The numbers of synapses shown are the numbers on any one neuron. The backprojection pathways for memory recall are shown in dashed green lines, and in red the CA3 recurrent collaterals via which ‘what’ and ‘where’ representations present at the same time can be associated during episodic memory storage, and via which completion of a whole memory from a part can occur during recall. All synapses are associatively modifiable except for the Dentate Gyrus (DG) mossy fiber (mf) synapses on the CA3 pyramidal cells. The dentate granule cells, the CA1 cells, and the entorhinal cortex inputs from the neocortex operate as competitive networks. The CA3 cells operate as an autoassociation attractor network to implement completion. The backprojection connections shown in green operate as pattern association networks for hippocampal system to neocortex memory recall (Rolls and Treves 2024; Rolls 2026). At the top, the central image shows what might be stored; the right image shows the recall cue, and the network then recalls in the Where neocortex where that face was in the scene shown on the left. This is what the network performs, and is typical of episodic memory in which the locations of faces or objects in a scene are remembered, in mental scene construction (from Rolls et al. 2024).

## Data Availability

No data were generated.
