# Supplementary material for: Mental Scene and Memory Construction and the Hippocampus
Source: Hippocampus. 2026 Sep 28;36(6):e70136. doi: 10.1002/hipo.70136 (PMC13620200; doi:10.1002/hipo.70136)
Supplement: Supplementary file 1 [file HIPO-36-0-s001.pdf]

Mental Scene and Memory Construction and the Hippocampus

Hippocampus (2026) doi: 10.1002/hipo.70136

Supplementary Material

Edmund T. Rolls[1, 2*]

1. Oxford Centre for Computational Neuroscience, Oxford, UK
2. Department of Computer Science, University of Warwick, Coventry, CV4 7AL, UK

*Corresponding author information:
Professor Edmund T. Rolls, MA, DPhil, DSc, Hon DSc
Department of Computer Science, University of Warwick, Coventry CV4 7AL, UK. Email: Edmund.Rolls@oxcns.org
URL: https://www.oxcns.org
https://orcid.org/0000-0003-3025-1292

Modified ordering of the HCP-MMP atlas

The atlas used to define brain regions was the HCP-MMP surface-based atlas (Glasser et al., 2016), illustrated in Fig. S1. In the HCP-MMP atlas, each region has its RegionID, which we show in Table S1. Detailed information about the regions is available in the Supplementary Material File NIHMS68870-supplement-Neuroanatomical_Supplementary_Results.pdf provided by Glasser et al (2016). In that Supplementary Material file, a grouping of the regions is suggested based on geographic proximity and functional similarities, and this grouping is shown in the column labelled CortexID in Table S1. That has led to a different ordering of the regions, which we show in Table S1, with the original regionIDs from the HCP atlas shown in the column headed 'regionID'. This reordered version of the HCP-MMP atlas is described by Dr Dianne Patterson of the University of Arizona at https://neuroimaging-core-docs.readthedocs.io/en/latest/pages/atlases.html, where the following supporting files used to help generate Table S1 are available: HCP-MMP_UniqueRegionList.csv and Glasser_2016_Table.xlsx. We made file HCPMMP_CortexID_Ordering.xlsx from this, and this is available from the present authors. The connectivity matrices shown in the present paper used the ordering shown in Table S1, which is also used in the volumetric and extended form of this atlas (Huang, Rolls, Feng, & Lin, 2022). An introduction to the regions in the HCP-MMP atlas is provided by Rolls (2026).

Table S1. Regions defined in the modified Human Connectome Project atlas (Glasser et al., 2016). L=left hemisphere, R=right. The column 'Reordered region ID' is that used in Figs. 1-5, and is a reordering of that based on suggestions in the Supplementary Information of Glasser et al (2016). In that Supplementary Information of that paper, the 360 regions are grouped based on geographic proximity and functional similarities, which was reorganized and provided by Dr Dianne Patterson of the University of Arizona at https://neuroimaging-core-docs.readthedocs.io/en/latest/pages/atlases.html with the HCP-MMP_UniqueRegionList.csv and is shown in the column labelled CortexID in Table S1. The volumes are in mm^3. This modified atlas with the reordering is described elsewhere (Huang et al., 2022).

Reordered ID (L, R)	Region	RegionLongName	Cortical Division	Cortex ID	Original ID	Voxel numbers (1mm^3) (L,R)
1, 181	V1	Primary_Visual_Cortex	Primary_Visual	1	1	13812, 13406
2, 182	V2	Second_Visual_Area	Early_Visual	2	4	9515, 9420
3, 183	V3	Third_Visual_Area	Early_Visual	2	5	7106, 7481
4, 184	V4	Fourth_Visual_Area	Early_Visual	2	6	4782, 4537
5, 185	IPS1	IntraParietal_Sulcus_Area_1	Dorsal_Stream_Visual	3	17	1751, 1750
6, 186	V3A	Area_V3A	Dorsal_Stream_Visual	3	13	2191, 2212
7, 187	V3B	Area_V3B	Dorsal_Stream_Visual	3	19	639, 731
8, 188	V6	Sixth_Visual_Area	Dorsal_Stream_Visual	3	3	1402, 1559
9, 189	V6A	Area_V6A	Dorsal_Stream_Visual	3	152	904, 734
10, 190	V7	Seventh_Visual_Area	Dorsal_Stream_Visual	3	16	1005, 1041
11, 191	FFC	Fusiform_Face_Complex	Ventral_Stream_Visual	4	18	3848, 4402
12, 192	PIT	Posterior_InferoTemporal_complex	Ventral_Stream_Visual	4	22	1392, 1386
13, 193	V8	Eighth_Visual_Area	Ventral_Stream_Visual	4	7	1361, 1175
14, 194	VMV1	VentroMedial_Visual_Area_1	Ventral_Stream_Visual	4	153	939, 1219
15, 195	VMV2	VentroMedial_Visual_Area_2	Ventral_Stream_Visual	4	160	639, 923
16, 196	VMV3	VentroMedial_Visual_Area_3	Ventral_Stream_Visual	4	154	941, 1242
17, 197	VVC	Ventral_Visual_Complex	Ventral_Stream_Visual	4	163	2487, 2753
18, 198	FST	Area_FST	MT+_Complex	5	157	1324, 1683
19, 199	LO1	Area_Lateral_Occipital_1	MT+_Complex	5	20	619, 909
20, 200	LO2	Area_Lateral_Occipital_2	MT+_Complex	5	21	1179, 1062
21, 201	LO3	Area_Lateral_Occipital_3	MT+_Complex	5	159	438, 915
22, 202	MST	Medial_Superior_Temporal_Area	MT+_Complex	5	2	794, 1036
23, 203	MT	Middle_Temporal_Area	MT+_Complex	5	23	620, 1005
24, 204	PH	Area_PH	MT+_Complex	5	138	3453, 3205
25, 205	V3CD	Area_V3CD	MT+_Complex	5	158	876, 1222
26, 206	V4t	Area_V4t	MT+_Complex	5	156	1037, 1249
27, 207	1	Area_1	SomaSens_Motor	6	51	6590, 5925
28, 208	2	Area_2	SomaSens_Motor	6	52	4278, 4727
29, 209	3a	Area_3a	SomaSens_Motor	6	53	2247, 2286
30, 210	3b	Primary_Sensory_Cortex	SomaSens_Motor	6	9	5451, 4350
31, 211	4	Primary_Motor_Cortex	SomaSens_Motor	6	8	10776, 10254
32, 212	23c	Area_23c	ParaCentral_MidCing	7	38	2259, 2498
33, 213	24dd	Dorsal_Area_24d	ParaCentral_MidCing	7	40	2665, 2820
34, 214	24dv	Ventral_Area_24d	ParaCentral_MidCing	7	41	1076, 1349
35, 215	5L	Area_5L	ParaCentral_MidCing	7	39	2249, 2327
36, 216	5m	Area_5m	ParaCentral_MidCing	7	36	1483, 2079
37, 217	5mv	Area_5m_ventral	ParaCentral_MidCing	7	37	1651, 1996
38, 218	6ma	Area_6m_anterior	ParaCentral_MidCing	7	44	3941, 4251
39, 219	6mp	Area_6mp	ParaCentral_MidCing	7	55	3701, 3105
40, 220	SCEF	Supplementary_and_Cingulate_Eye_Field	ParaCentral_MidCing	7	43	3500, 3371

41, 221	55b	Area_55b	Premotor	8	12	2422, 1537
42, 222	6a	Area_6_anterior	Premotor	8	96	4233, 3752
43, 223	6d	Dorsal_area_6	Premotor	8	54	2916, 2909
44, 224	6r	Rostral_Area_6	Premotor	8	78	3029, 3981
45, 225	6v	Ventral_Area_6	Premotor	8	56	2075, 2516
46, 226	FEF	Frontal_Eye_Fields	Premotor	8	10	1787, 1889
47, 227	PEF	Premotor_Eye_Field	Premotor	8	11	1006, 1258
48, 228	43	Area_43	Posterior_Opercular	9	99	1889, 1678
49, 229	FOP1	Frontal_Opercular_Area_1	Posterior_Opercular	9	113	879, 932
50, 230	OP1	Area_OP1-SII	Posterior_Opercular	9	101	1275, 1072
51, 231	OP2-3	Area_OP2-3-VS	Posterior_Opercular	9	102	943, 792
52, 232	OP4	Area_OP4-PV	Posterior_Opercular	9	100	2332, 2409
53, 233	52	Area_52	Early_Auditory	10	103	725, 580
54, 234	A1	Primary_Auditory_Cortex	Early_Auditory	10	24	1023, 796
55, 235	LBelt	Lateral_Belt_Complex	Early_Auditory	10	174	820, 901
56, 236	MBelt	Medial_Belt_Complex	Early_Auditory	10	173	1242, 1236
57, 237	PBelt	ParaBelt_Complex	Early_Auditory	10	124	1719, 1439
58, 238	PFcm	Area_PFcm	Early_Auditory	10	105	1486, 1485
59, 239	RI	RetroInsular_Cortex	Early_Auditory	10	104	1149, 1334
60, 240	A4	Auditory_4_Complex	Auditory_Association	11	175	3514, 3610
61, 241	A5	Auditory_5_Complex	Auditory_Association	11	125	3346, 3881
62, 242	STGa	Area_STGa	Auditory_Association	11	123	2509, 2187
63, 243	STSda	Area_STSd_anterior	Auditory_Association	11	128	1944, 2389
64, 244	STSdp	Area_STSd_posterior	Auditory_Association	11	129	1994, 2605
65, 245	STSva	Area_STSv_anterior	Auditory_Association	11	176	1694, 1900
66, 246	STSvp	Area_STSv_posterior	Auditory_Association	11	130	2898, 2515
67, 247	TA2	Area_TA2	Auditory_Association	11	107	1518, 1726
68, 248	AAIC	Anterior_Agranular_Insula_Complex	Insula_FrontalOperc	12	112	1859, 1691
69, 249	AVI	Anterior_Ventral_Insular_Area	Insula_FrontalOperc	12	111	1446, 1792
70, 250	FOP2	Frontal_Opercular_Area_2	Insula_FrontalOperc	12	115	750, 720
71, 251	FOP3	Frontal_Opercular_Area_3	Insula_FrontalOperc	12	114	754, 614
72, 252	FOP4	Frontal_Opercular_Area_4	Insula_FrontalOperc	12	108	2522, 1678
73, 253	FOP5	Area_Frontal_Opercular_5	Insula_FrontalOperc	12	169	1297, 1365
74, 254	Ig	Insular_Granular_Complex	Insula_FrontalOperc	12	168	841, 1077
75, 255	MI	Middle_Insular_Area	Insula_FrontalOperc	12	109	2102, 1960
76, 256	PI	Para-Insular_Area	Insula_FrontalOperc	12	178	1033, 1058
77, 257	Pir	Piriform_Cortex	Insula_FrontalOperc	12	110	2287, 1856
78, 258	PoI1	Area_Posterior_Insular_1	Insula_FrontalOperc	12	167	1811, 1835
79, 259	PoI2	Posterior_Insular_Area_2	Insula_FrontalOperc	12	106	2747, 2675
80, 260	H	Hippocampus	Medial_Temporal	13	120	4283, 3626
81, 261	PreS	PreSubiculum	Medial_Temporal	13	119	1817, 1558
82, 262	EC	Entorhinal_Cortex	Medial_Temporal	13	118	2127, 2110
83, 263	PeEc	Perirhinal_Ectorhinal_Cortex	Medial_Temporal	13	122	4826, 4755
84, 264	TF	Area_TF	Medial_Temporal	13	135	3986, 4752
85, 265	PHA1	ParaHippocampal_Area_1	Medial_Temporal	13	126	1281, 1168
86, 266	PHA2	ParaHippocampal_Area_2	Medial_Temporal	13	155	783, 771
87, 267	PHA3	ParaHippocampal_Area_3	Medial_Temporal	13	127	2023, 1122
88, 268	PHT	Area_PHT	Lateral_Temporal	14	137	4182, 3410
89, 269	TE1a	Area_TE1_anterior	Lateral_Temporal	14	132	5227, 4180
90, 270	TE1m	Area_TE1_Middle	Lateral_Temporal	14	177	3339, 3429

91, 271	TE1p	Area_TE1_posterior	Lateral_Temporal	14	133	7116, 6010
92, 272	TE2a	Area_TE2_anterior	Lateral_Temporal	14	134	5691, 5753
93, 273	TE2p	Area_TE2_posterior	Lateral_Temporal	14	136	4115, 3040
94, 274	TGd	Area_TG_dorsal	Lateral_Temporal	14	131	10192, 10269
95, 275	TGv	Area_TG_Ventral	Lateral_Temporal	14	172	3694, 4515
96, 276	PSL	PeriSylvian_Language_Area	TPO	15	25	2154, 2759
97, 277	STV	Superior_Temporal_Visual_Area	TPO	15	28	2322, 2294
98, 278	TPOJ1	Area_TemporoParietoOccipital_Junction_1	TPO	15	139	2102, 3938
99, 279	TPOJ2	Area_TemporoParietoOccipital_Junction_2	TPO	15	140	1930, 2068
100, 280	TPOJ3	Area_TemporoParietoOccipital_Junction_3	TPO	15	141	1290, 1277
101, 281	7AL	Lateral_Area_7A	Superior_Parietal	16	42	2134, 2030
102, 282	7Am	Medial_Area_7A	Superior_Parietal	16	45	2995, 2379
103, 283	7PC	Area_7PC	Superior_Parietal	16	47	3151, 3415
104, 284	7PL	Lateral_Area_7P	Superior_Parietal	16	46	1695, 1363
105, 285	7Pm	Medial_Area_7P	Superior_Parietal	16	29	1601, 1308
106, 286	AIP	Anterior_IntraParietal_Area	Superior_Parietal	16	117	1999, 2542
107, 287	LIPd	Area_Lateral_IntraParietal_dorsal	Superior_Parietal	16	95	1008, 869
108, 288	LIPv	Area_Lateral_IntraParietal_ventral	Superior_Parietal	16	48	1681, 1783
109, 289	MIP	Medial_IntraParietal_Area	Superior_Parietal	16	50	1872, 2403
110, 290	VIP	Ventral_IntraParietal_Complex	Superior_Parietal	16	49	1890, 1577
111, 291	IP0	Area_IntraParietal_0	Inferior_Parietal	17	146	1203, 1239
112, 292	IP1	Area_IntraParietal_1	Inferior_Parietal	17	145	1692, 1632
113, 293	IP2	Area_IntraParietal_2	Inferior_Parietal	17	144	2102, 1861
114, 294	PF	Area_PF_Complex	Inferior_Parietal	17	148	5457, 5251
115, 295	PFm	Area_PFm_Complex	Inferior_Parietal	17	149	8220, 8141
116, 296	PFop	Area_PF_Opercular	Inferior_Parietal	17	147	1797, 1783
117, 297	PFt	Area_PFt	Inferior_Parietal	17	116	1983, 2039
118, 298	PGi	Area_PGi	Inferior_Parietal	17	150	4791, 4970
119, 299	PGp	Area_PGp	Inferior_Parietal	17	143	2501, 3740
120, 300	PGs	Area_PGs	Inferior_Parietal	17	151	4552, 3366
121, 301	23d	Area_23d	Posterior_Cingulate	18	32	1261, 1513
122, 302	31a	Area_31a	Posterior_Cingulate	18	162	1260, 1116
123, 303	31pd	Area_31pd	Posterior_Cingulate	18	161	1428, 864
124, 304	31pv	Area_31p_ventral	Posterior_Cingulate	18	35	950, 1022
125, 305	7m	Area_7m	Posterior_Cingulate	18	30	2128, 2067
126, 306	d23ab	Area_dorsal_23_a+b	Posterior_Cingulate	18	34	1607, 1106
127, 307	DVT	Dorsal_Transitional_Visual_Area	Posterior_Cingulate	18	142	1806, 2176
128, 308	PCV	PreCuneus_Visual_Area	Posterior_Cingulate	18	27	2245, 2416
129, 309	POS1	Parieto-Occipital_Sulcus_Area_1	Posterior_Cingulate	18	31	2531, 2727
130, 310	POS2	Parieto-Occipital_Sulcus_Area_2	Posterior_Cingulate	18	15	3261, 3093
131, 311	ProS	ProStriate_Area	Posterior_Cingulate	18	121	1222, 1055
132, 312	RSC	RetroSplenial_Complex	Posterior_Cingulate	18	14	2830, 3067
133, 313	v23ab	Area_ventral_23_a+b	Posterior_Cingulate	18	33	916, 1089
134, 314	10r	Area_10r	AntCing_MedPFC	19	65	1589, 1053
135, 315	10v	Area_10v	AntCing_MedPFC	19	88	3906, 2667
136, 316	25	Area_25	AntCing_MedPFC	19	164	1911, 2135
137, 317	33pr	Area_33_prime	AntCing_MedPFC	19	58	1354, 1316
138, 318	8BM	Area_8BM	AntCing_MedPFC	19	63	3122, 3436
139, 319	9m	Area_9_Middle	AntCing_MedPFC	19	69	6338, 5881

140, 320	a24	Area_a24	AntCing_MedPFC	19	61	2085, 2152
141, 321	a24pr	Anterior_24_prime	AntCing_MedPFC	19	59	1095, 1474
142, 322	a32pr	Area_anterior_32_prime	AntCing_MedPFC	19	179	1759, 1118
143, 323	d32	Area_dorsal_32	AntCing_MedPFC	19	62	2228, 2374
144, 324	p24	Area_posterior_24	AntCing_MedPFC	19	180	2394, 2442
145, 325	p24pr	Area_Posterior_24_prime	AntCing_MedPFC	19	57	1422, 1724
146, 326	p32	Area_p32	AntCing_MedPFC	19	64	1180, 1765
147, 327	p32pr	Area_p32_prime	AntCing_MedPFC	19	60	1569, 1305
148, 328	pOFC	Posterior_OFC_Complex	AntCing_MedPFC	19	166	2486, 2836
149, 329	s32	Area_s32	AntCing_MedPFC	19	165	604, 1015
150, 330	10d	Area_10d	OrbPolaFrontal	20	72	3644, 3096
151, 331	10pp	Polar_10p	OrbPolaFrontal	20	90	1997, 2487
152, 332	11l	Area_11l	OrbPolaFrontal	20	91	3531, 3793
153, 333	13l	Area_13l	OrbPolaFrontal	20	92	2429, 1757
154, 334	47m	Area_47m	OrbPolaFrontal	20	66	799, 781
155, 335	47s	Area_47s	OrbPolaFrontal	20	94	2795, 3080
156, 336	a10p	Area_anterior_10p	OrbPolaFrontal	20	89	1964, 1748
157, 337	OFC	Orbital_Frontal_Complex	OrbPolaFrontal	20	93	4560, 5232
158, 338	p10p	Area_posterior_10p	OrbPolaFrontal	20	170	2116, 2365
159, 339	44	Area_44	Inferior_Frontal	21	74	2435, 2589
160, 340	45	Area_45	Inferior_Frontal	21	75	3762, 2962
161, 341	47l	Area_47l_(47_lateral)	Inferior_Frontal	21	76	2527, 2592
162, 342	a47r	Area_anterior_47r	Inferior_Frontal	21	77	4167, 3763
163, 343	IFJa	Area_IFJa	Inferior_Frontal	21	79	1513, 1405
164, 344	IFJp	Area_IFJp	Inferior_Frontal	21	80	960, 740
165, 345	IFSa	Area_IFSa	Inferior_Frontal	21	82	2057, 2641
166, 346	IFSp	Area_IFSp	Inferior_Frontal	21	81	1589, 1730
167, 347	p47r	Area_posterior_47r	Inferior_Frontal	21	171	2133, 1761
168, 348	46	Area_46	Dorsolateral_Prefrontal	22	84	4863, 4394
169, 349	8Ad	Area_8Ad	Dorsolateral_Prefrontal	22	68	3386, 3492
170, 350	8Av	Area_8Av	Dorsolateral_Prefrontal	22	67	4807, 5902
171, 351	8BL	Area_8B_Lateral	Dorsolateral_Prefrontal	22	70	3377, 4078
172, 352	8C	Area_8C	Dorsolateral_Prefrontal	22	73	4085, 3134
173, 353	9-46d	Area_9-46d	Dorsolateral_Prefrontal	22	86	4534, 4666
174, 354	9a	Area_9_anterior	Dorsolateral_Prefrontal	22	87	3706, 3048
175, 355	9p	Area_9_Posterior	Dorsolateral_Prefrontal	22	71	3426, 2488
176, 356	a9-46v	Area_anterior_9-46v	Dorsolateral_Prefrontal	22	85	3314, 2628
177, 357	i6-8	Inferior_6-8_Transitional_Area	Dorsolateral_Prefrontal	22	97	1764, 2418
178, 358	p9-46v	Area_posterior_9-46v	Dorsolateral_Prefrontal	22	83	2871, 4635
179, 359	s6-8	Superior_6-8_Transitional_Area	Dorsolateral_Prefrontal	22	98	1336, 2132
180, 360	SFL	Superior_Frontal_Language_Area	Dorsolateral_Prefrontal	22	26	3873, 3055

Column 1 (Reordered ID) shows the order in HCPex based on the HCP-MMP1_UniqueRegionList.csv, as described in the Methods, of the 360 cortical regions originally defined by Glasser et al (2016). The names of the cortical divisions shown in column 4 come from the same .csv file. The sixth column shows the original order used by Glasser et al (2016). Abbreviations: L=left hemisphere, R=right. MT+_Complex, MT+_Complex_and_Neighboring_Visual_Areas; SomaSens_Motor, Somatosensory_and_Motor; ParaCentral_MidCing, Paracentral_Lobular_and_Mid_Cingulate; Insula_FrontalOperc, Insular_and_Frontal_Opercular; TPO, Temporo-Parieto-Occipital_Junction; AntCing_MedPFC, Anterior_Cingulate_and_Medial_Prefrontal; OrbPolaFrontal, Orbital_and_Polar_Frontal.

Fig. S1-1. Example coronal slices showing regions defined in the HCPex atlas and added subcortical regions (Huang et al., 2022). The abbreviations are as in Table S1. The y values for the coronal slices are in MNI coordinates.

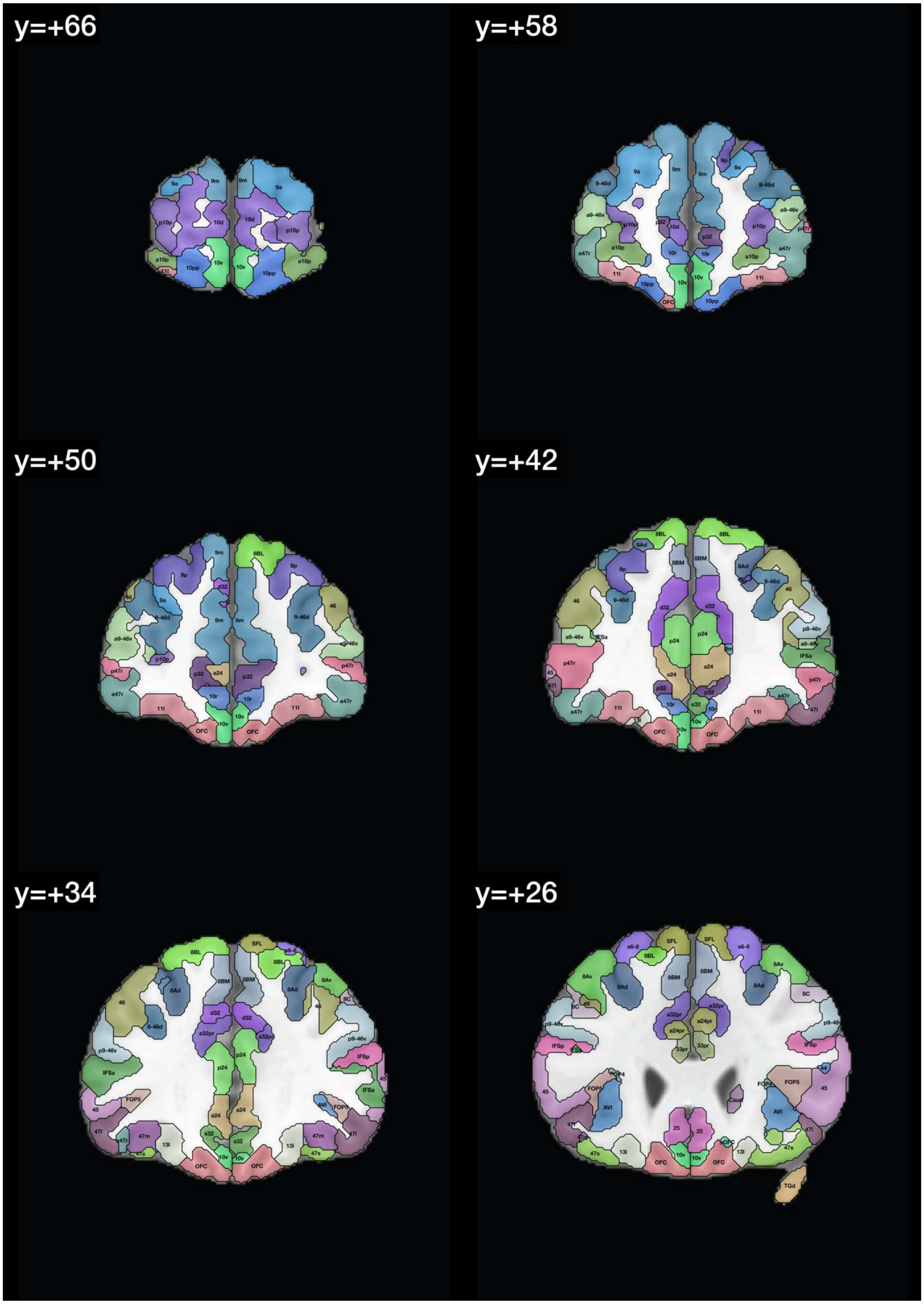

Fig. S1-2. Example coronal slices showing regions defined in the HCPex atlas and added subcortical regions (Huang et al., 2022). The abbreviations are as in Table S1. The y values for the coronal slices are in MNI coordinates.

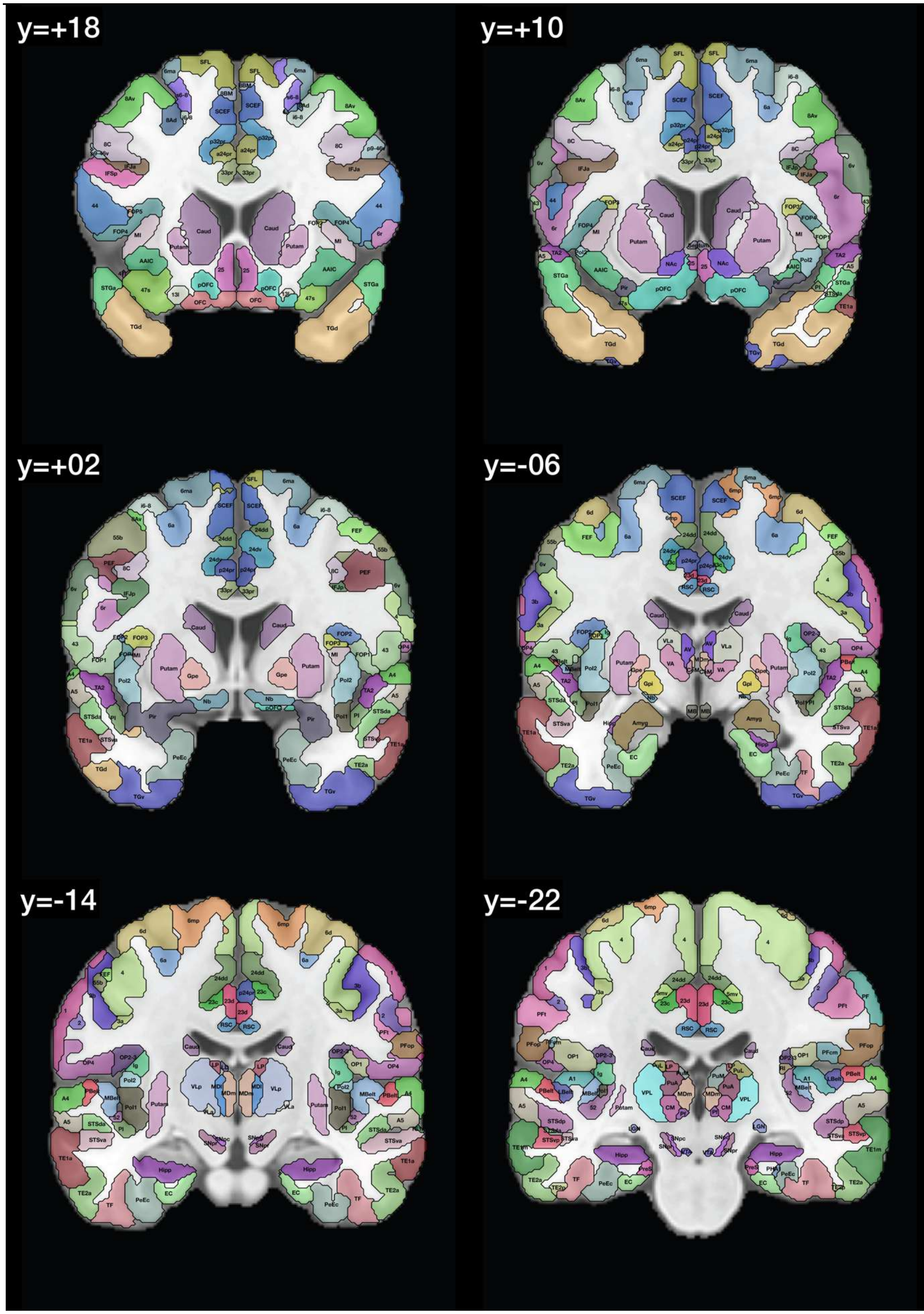

Fig. S1-3. Example coronal slices showing regions defined in the HCPex atlas and added subcortical regions (Huang et al., 2022). The abbreviations are as in Table S1. The y values for the coronal slices are in MNI coordinates.

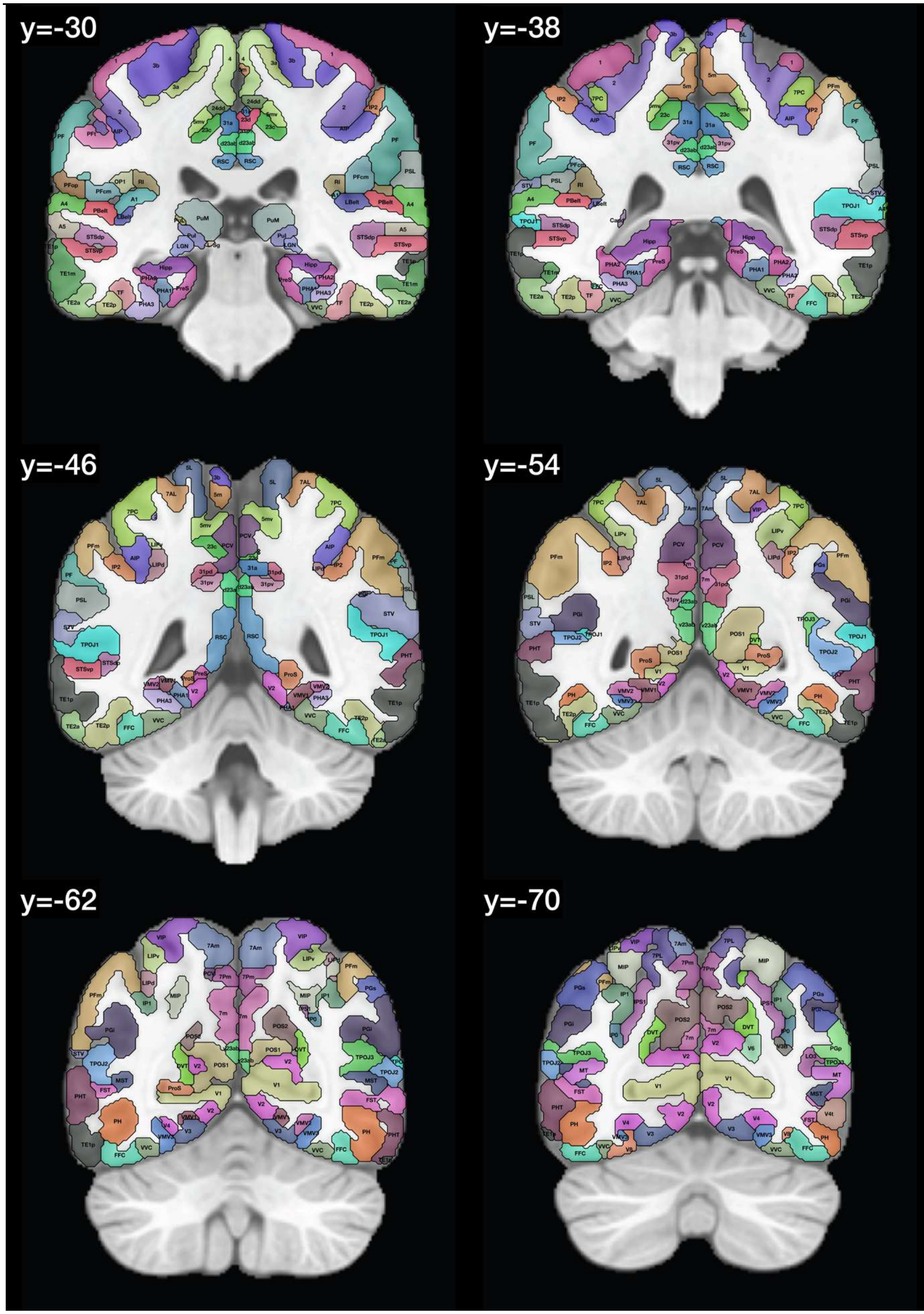

Fig. S1-4. Example coronal slices showing regions defined in the HCPex atlas and added subcortical regions (Huang et al., 2022). The abbreviations are as in Table S1. The y values for the coronal slices are in MNI coordinates.

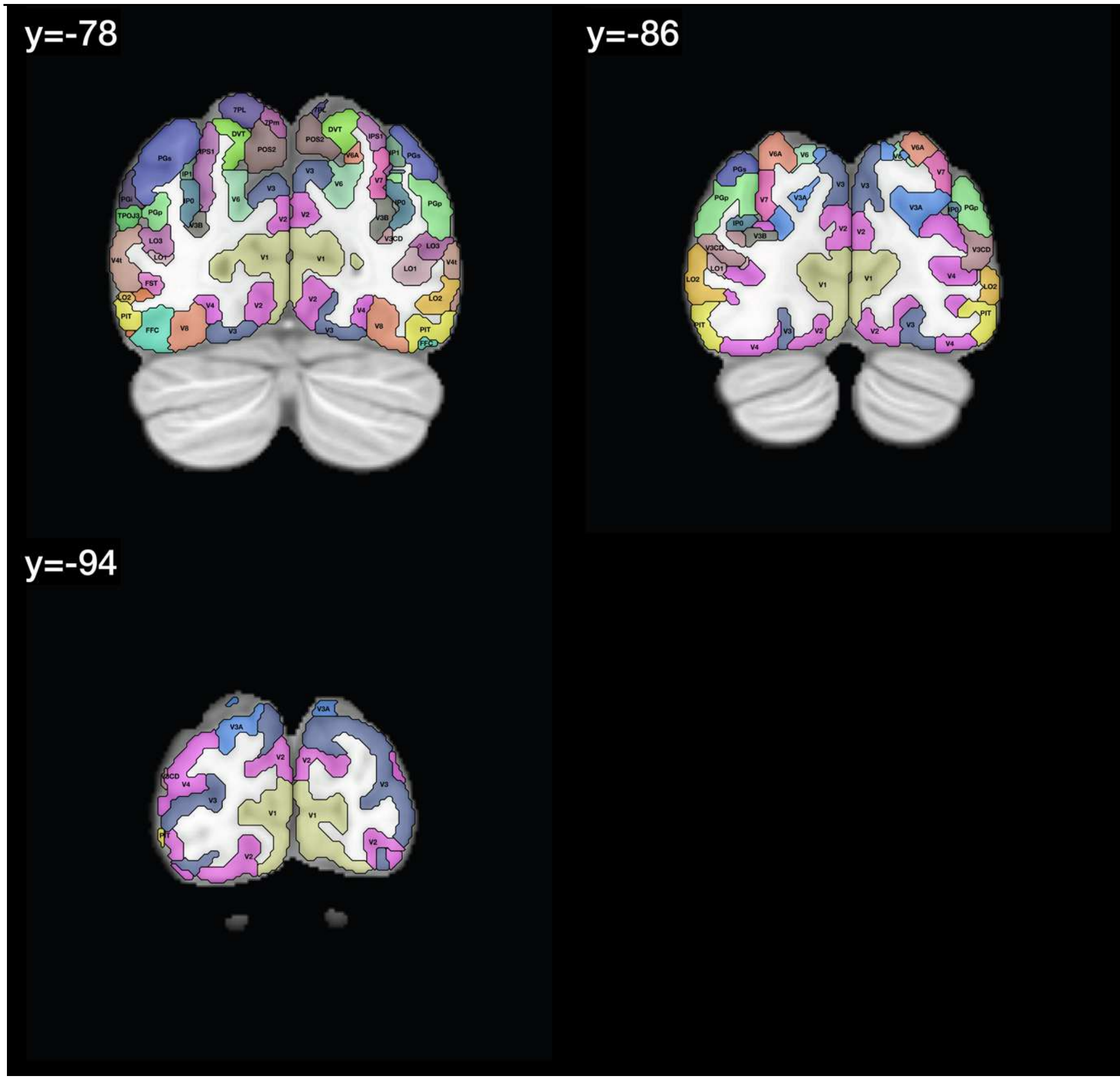
y=-78
y=-86
y=-94

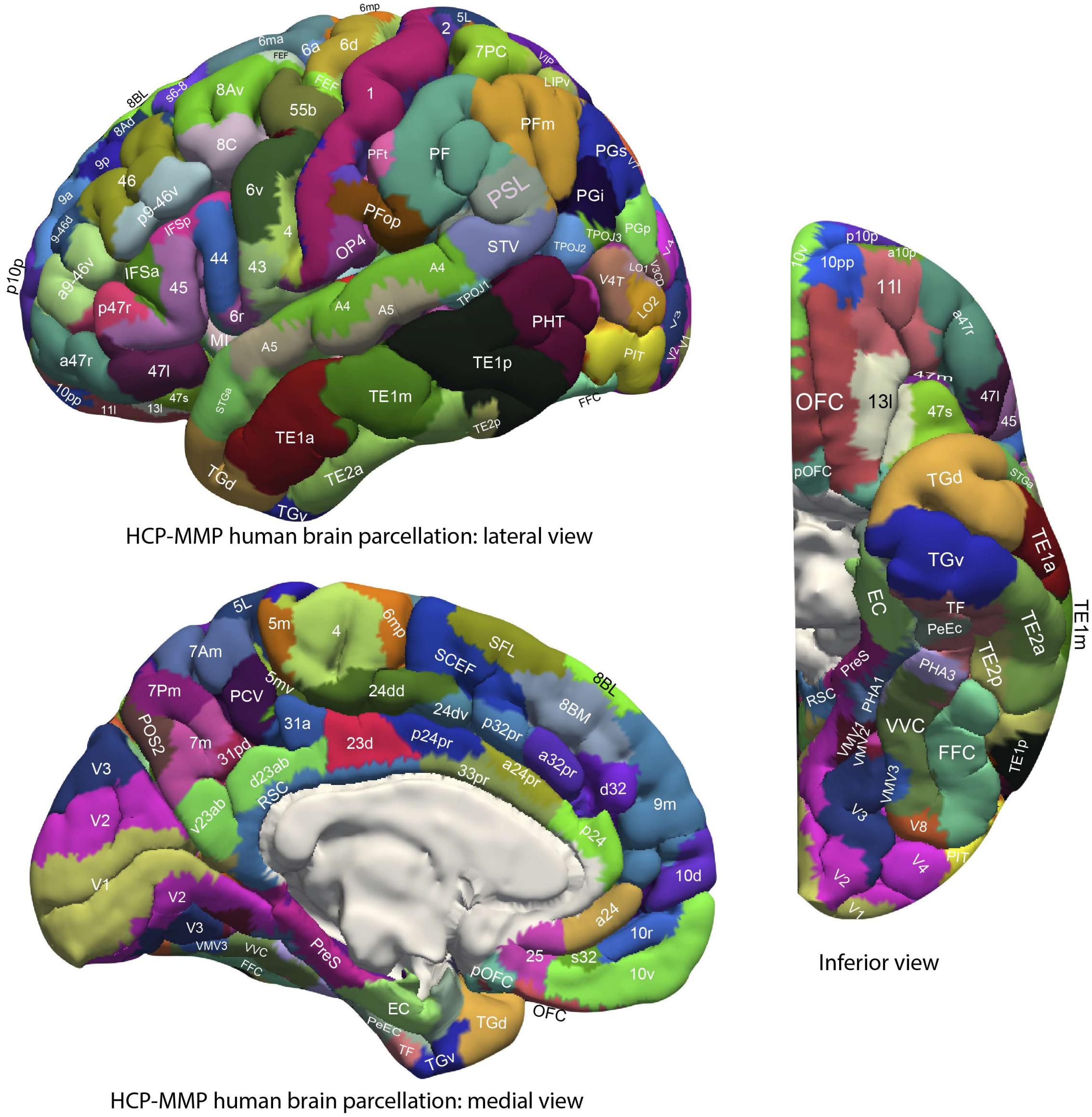

Fig. S1-5. Anatomical regions of the human visual and other cortical regions. Regions are shown as defined in the HCP-MMP atlas (Glasser et al., 2016), and in its extended version HCPex (Huang et al., 2022). The regions are shown on images of the human brain without the sulci expanded to show which cortical HCP-MMP regions are normally visible. (The ICBM153 MNI T1 image was used to prepare this figure.) Abbreviations are provided in Table S1.

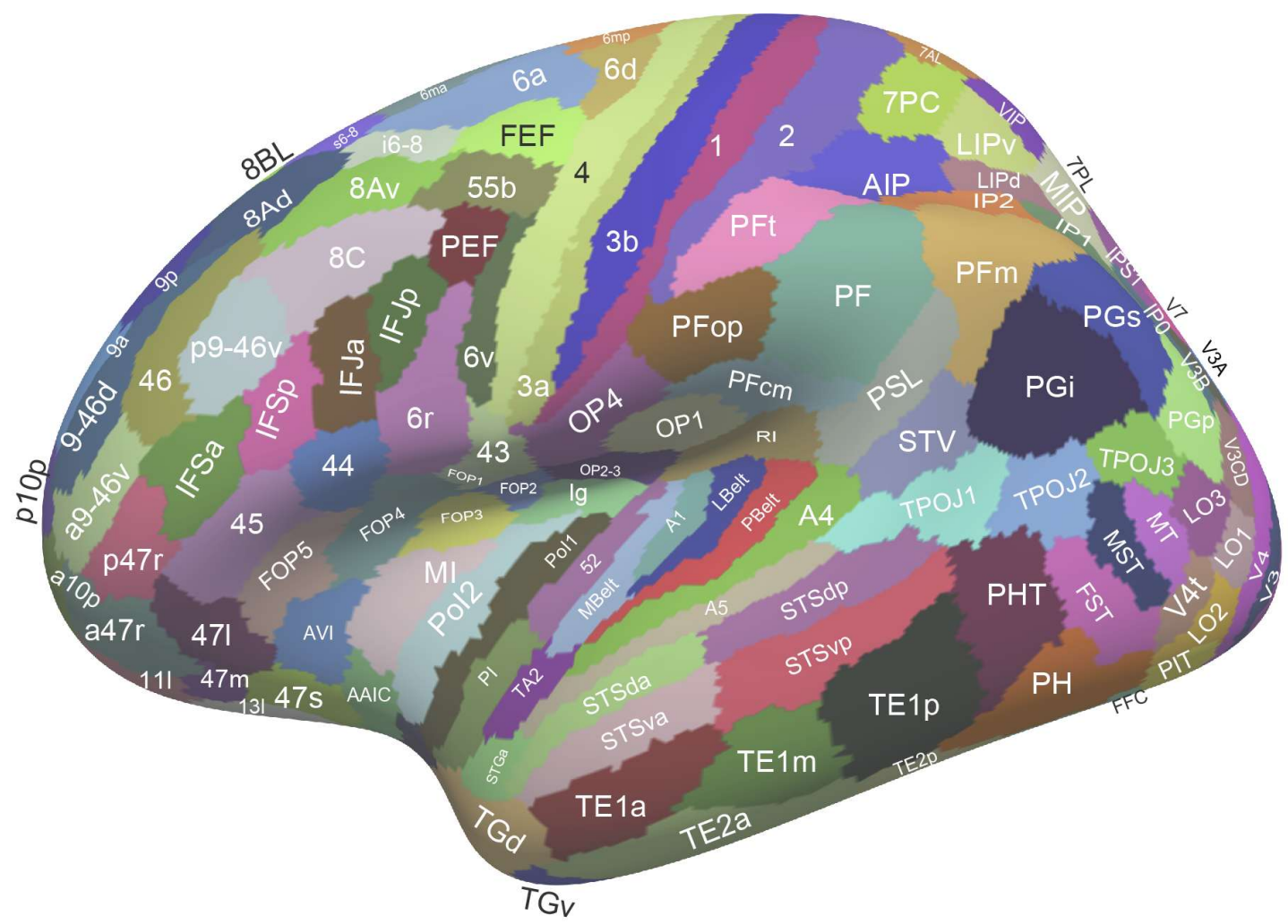

HCP-MMP1 human brain parcellation: lateral view

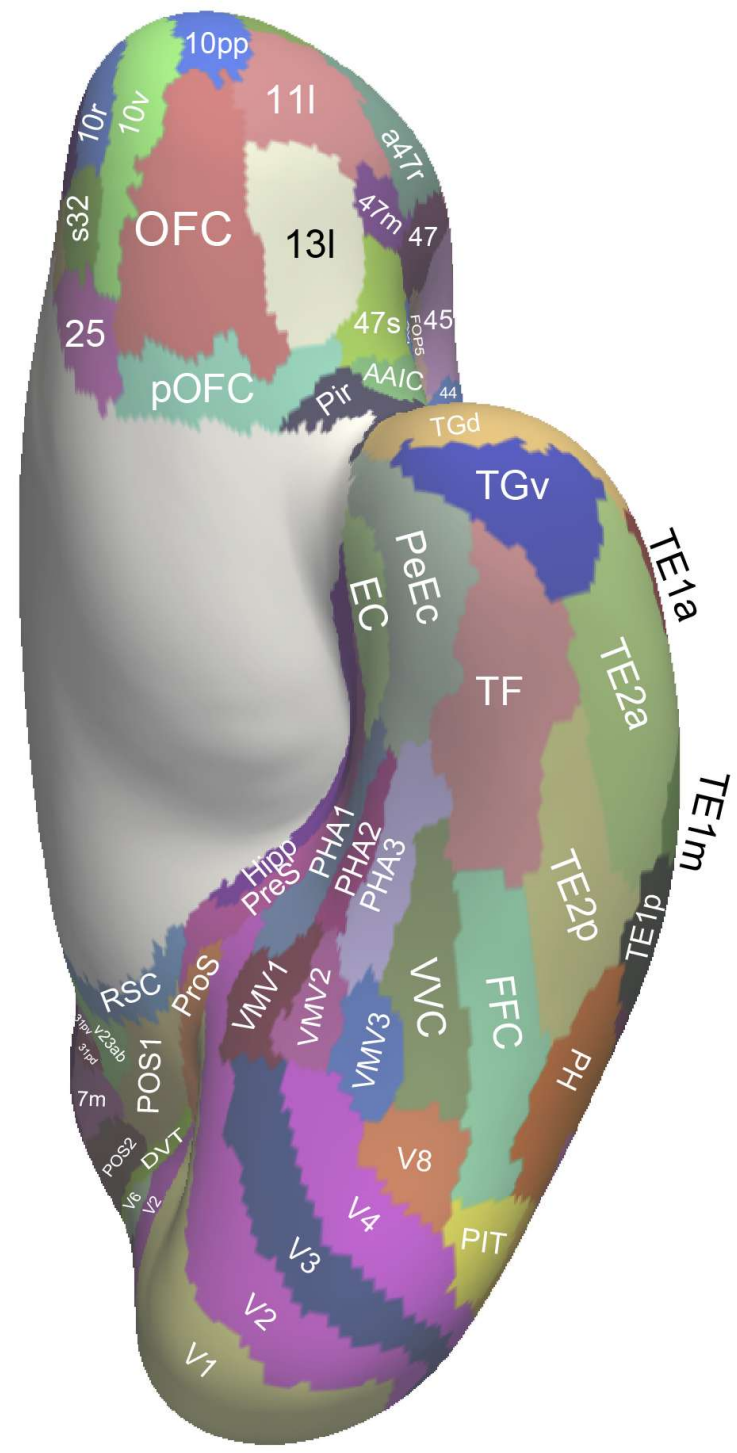

Inferior view

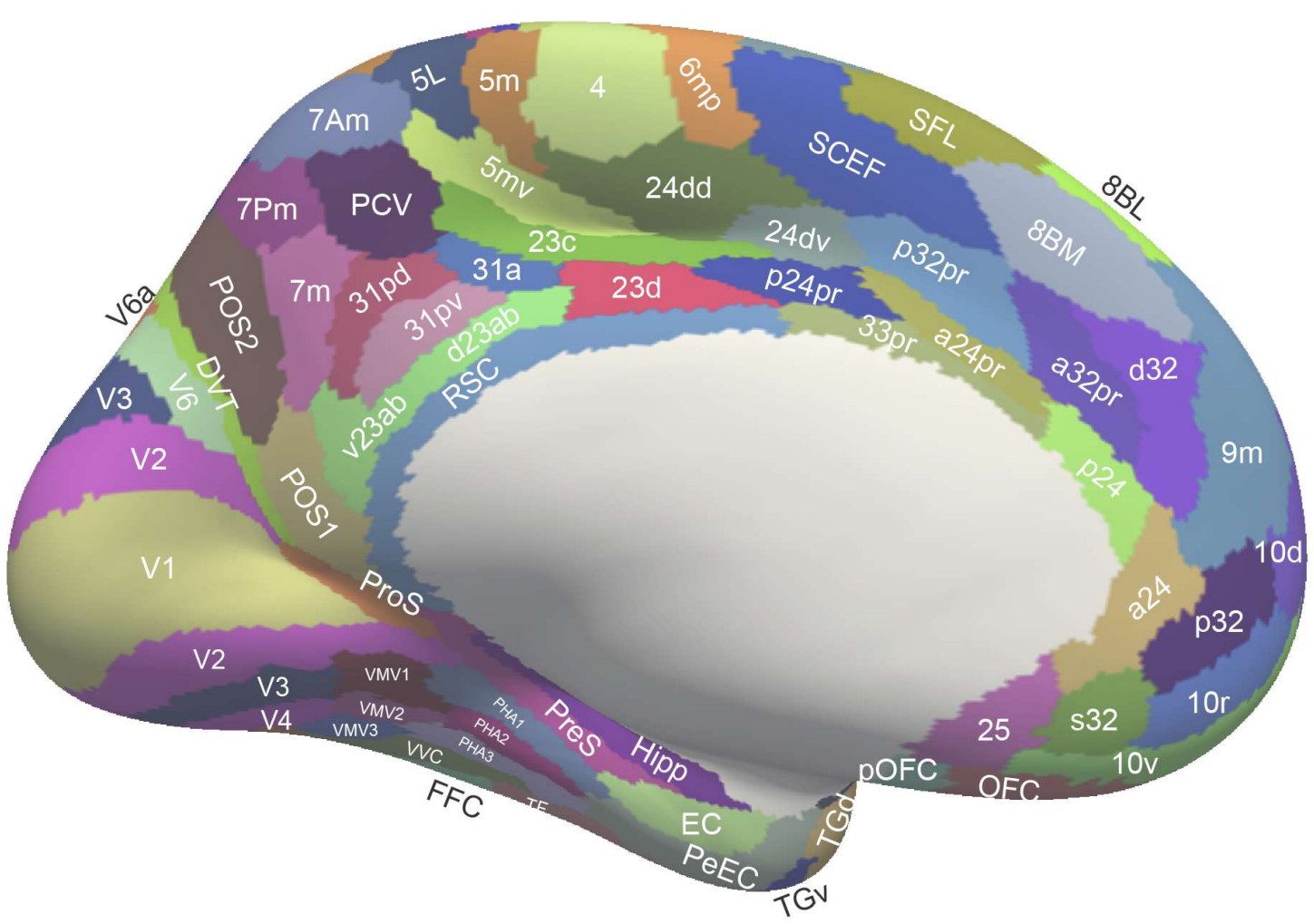

HCP-MMP1 human brain parcellation: medial view

Fig. S1-6. Regions in the Human Connectome Project Multimodal Parcellation atlas (HCP-MMP) (Glasser et al., 2016) and its extended version HCPex (Huang et al., 2022) to show the cortical regions. The regions are shown on images of the human brain with the sulci expanded sufficiently to allow the regions within the sulci to be shown. Abbreviations are provided in Table S1. For comparison, a version of this diagram without the sulci expanded is provided in Fig. S1-5. (HCPBrainMaster4bLC.eps)

The neocortex-hippocampus-neocortex model

The architecture of the neocortex-hippocampus-neocortex integrate-and-fire model of the recall of the whole neocortical what-where memory referred to in Section 10.2 of the paper is shown in Fig. S2. Details of the operation of the model are provided elsewhere (Rolls, Zhang, & Feng, 2024).

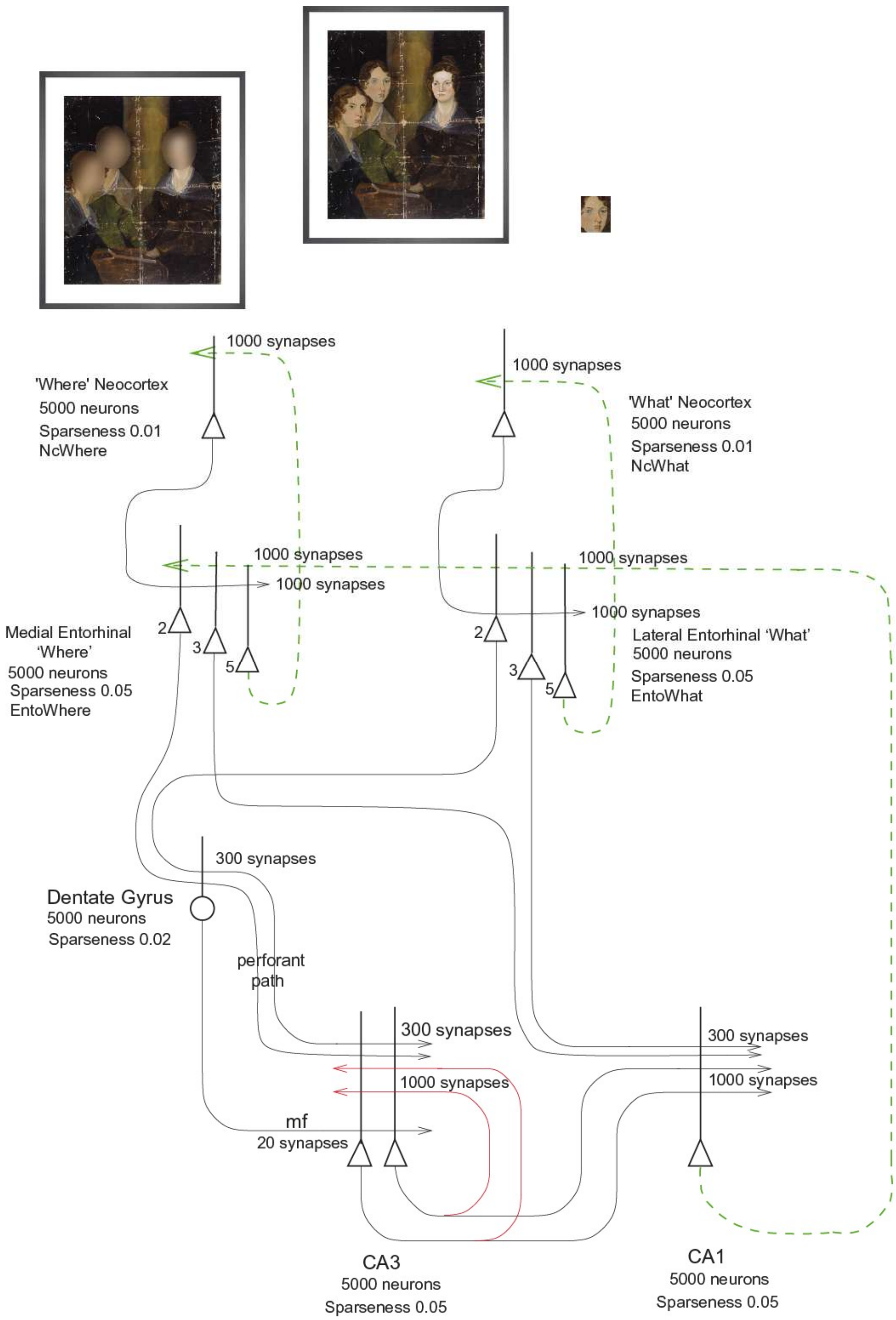

Fig. S2. Simulation of neocortical 'what' and 'where' inputs to the hippocampus for the storage of episodic memory, and for the recall of 'what' (object or face) and 'where' (spatial view) information back to the 'what' and 'where' neocortex. The pyramidal cell bodies are shown as triangles, the dendrites as the thick lines above the cell bodies, and the axons as thin lines terminated with an arrow. The numbers of synapses shown are the numbers on any one neuron. The backprojection pathways for memory recall are shown in dashed green lines, and in red the CA3

recurrent collaterals via which 'what' and 'where' representations present at the same time can be associated during episodic memory storage, and via which completion of a whole memory from a part can occur during recall. All synapses are associatively modifiable except for the Dentate Gyrus (DG) mossy fibre (mf) synapses on the CA3 pyramidal cells. The dentate granule cells, the CA1 cells, and the entorhinal cortex inputs from the neocortex operate as competitive networks. The CA3 cells operate as an autoassociation attractor network to implement completion. The backprojection connections shown in green operate as pattern association networks for hippocampal system to neocortex memory recall (Rolls & Treves, 2024; Rolls, 2026). At the top, the central image shows what might be stored; the right image shows the recall cue, and the network then recalls in the Where neocortex where that face was in the scene shown on the left. This is what the network performs, and is typical of episodic memory in which the locations of faces or objects in a scene are remembered, in mental scene construction. (From Rolls et al (2024)) (NChippArch24Images3.eps)

References

Glasser, M. F., Coalson, T. S., Robinson, E. C., Hacker, C. D., Harwell, J., Yacoub, E., . . . Van Essen, D. C. (2016). A multi-modal parcellation of human cerebral cortex. *Nature, 536*, 171-178.

Huang, C. C., Rolls, E. T., Feng, J., & Lin, C. P. (2022). An extended Human Connectome Project multimodal parcellation atlas of the human cortex and subcortical areas. *Brain Struct Funct, 227*, 763-778.

Rolls, E. T., & Treves, A. (2024). A theory of hippocampal function: New developments. *Progress in Neurobiology, 238*, 102636.

Rolls, E. T., Zhang, C., & Feng, J. (2024). Hippocampal storage and recall of neocortical 'What' - 'Where' representations. *Hippocampus, 34*, 608-624.

Rolls, E. T. (2026). *Brain Computations and Principles; and AI*. Oxford: Oxford University Press, Open Access.
